# Rapid and selective generation of H_2_S within mitochondria protects against cardiac ischemia-reperfusion injury

**DOI:** 10.1016/j.redox.2022.102429

**Published:** 2022-08-05

**Authors:** Jan Lj. Miljkovic, Nils Burger, Justyna M. Gawel, John F. Mulvey, Abigail A.I. Norman, Takanori Nishimura, Yoshiyuki Tsujihata, Angela Logan, Olga Sauchanka, Stuart T. Caldwell, Jordan L. Morris, Tracy A. Prime, Stefan Warrington, Julien Prudent, Georgina R. Bates, Dunja Aksentijević, Hiran A. Prag, Andrew M. James, Thomas Krieg, Richard C. Hartley, Michael P. Murphy

**Affiliations:** a MRC Mitochondrial Biology Unit, University of Cambridge, Cambridge Biomedical Campus, CB2 0XY, UK; b School of Chemistry, University of Glasgow, Glasgow, G12 8QQ, UK; c Department of Medicine, University of Cambridge, Cambridge, CB2 0QQ, UK; d Innovative Biology Laboratories, Neuroscience Drug Discovery Unit, Takeda Pharmaceutical Company Limited, 251-8555, Japan; e Centre for Biochemical Pharmacology, William Harvey Research Institute, Barts and the London School of Medicine and Dentistry, Queen Mary University of London, Charterhouse Square, London, United Kingdom

**Keywords:** Hydrogen sulfide donors, Mitochondria, Ischemia-reperfusion injury, Mitochondria targeting, Reverse electron transport (RET)

## Abstract

Mitochondria-targeted H_2_S donors are thought to protect against acute ischemia-reperfusion (IR) injury by releasing H_2_S that decreases oxidative damage. However, the rate of H_2_S release by current donors is too slow to be effective upon administration following reperfusion. To overcome this limitation here we develop a mitochondria-targeted agent, MitoPerSulf that very rapidly releases H_2_S within mitochondria. MitoPerSulf is quickly taken up by mitochondria, where it reacts with endogenous thiols to generate a persulfide intermediate that releases H_2_S. MitoPerSulf is acutely protective against cardiac IR injury in mice, due to the acute generation of H_2_S that inhibits respiration at cytochrome *c* oxidase thereby preventing mitochondrial superoxide production by lowering the membrane potential. Mitochondria-targeted agents that rapidly generate H_2_S are a new class of therapy for the acute treatment of IR injury.

## 1 Introduction

Hydrogen sulfide (H_2_S) and H_2_S releasing compounds are protective against ischemia-reperfusion (IR) injury [[1], [2], [3]] in the liver [4,5], kidney [6], lung [7] and heart [[8], [9], [10], [11]] and against IR injury during organ transplantation [12,13]. The H_2_S donors used so far include simple hydrosulfide, disulfide and trisulfide salts that spontaneously hydrolyse to release H_2_S [[14], [15], [16]], as well as H_2_S donors such as GYY 4137 [[17], [18], [19]], HS-NSAIDs [20], S-diclofenac [21], DATS-MSN [22] and ammonium tetrathiomolybdate [23].

The production of superoxide by the mitochondrial respiratory chain upon reperfusion of ischemic tissue is a key initiator of the oxidative damage that underlies IR injury [[24], [25], [26]]. Consequently, there is considerable interest in developing H_2_S-donors that protect against IR injury by decreasing mitochondrial oxidative damage [[27], [28], [29], [30]]. Candidate protective mechanisms include free-radical scavenging by H_2_S [[31], [32], [33], [34]] or via the reversible *S*-thiolation of protein cysteine residues to form a persulfide (r-SPSH) [35,36] that can prevent irreversible oxidative damage to cysteine residues and may enhance the protective activity of some proteins [37,38]. Alternatively, H_2_S is a reversible inhibitor of cytochrome *c* oxidase [39]. Thereby, H_2_S may lower the proton motive force, a major driver of mitochondrial superoxide production upon reperfusion following ischemia [24,25], but whether this contributes to its protection against IR injury is not known.

**Fig. 1:**
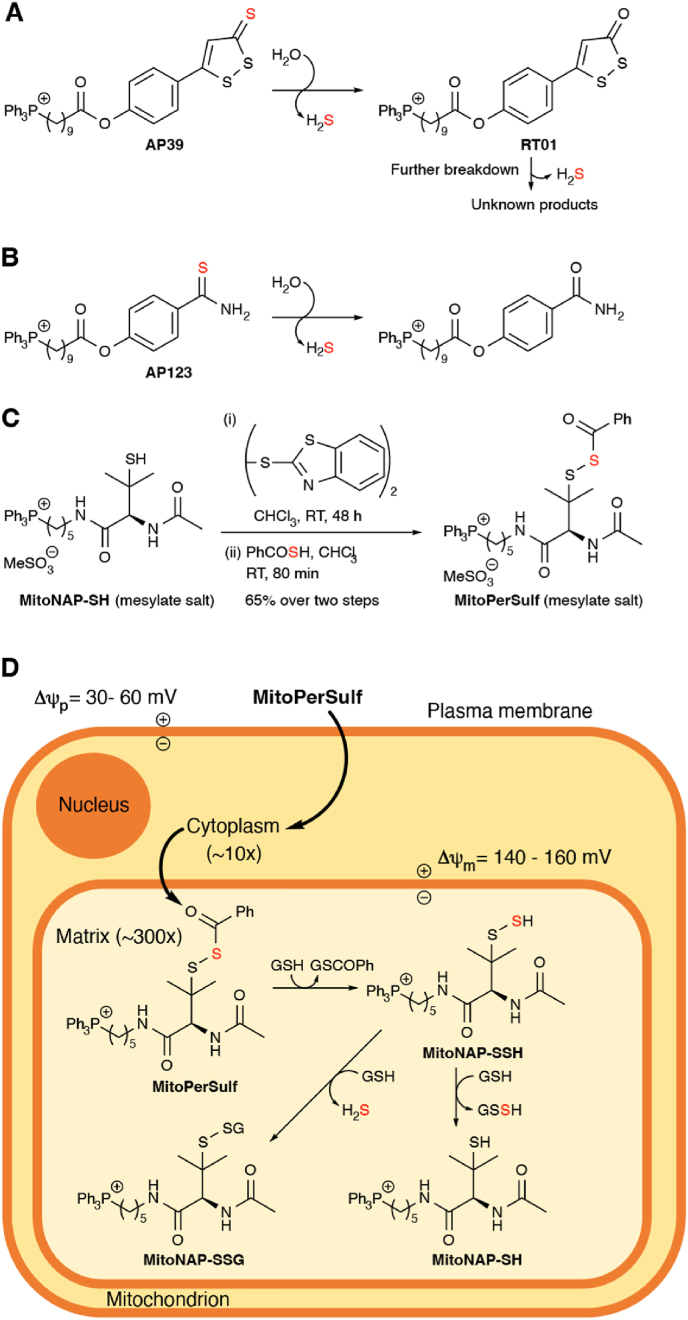
Mitochondria-Targeted H_2_S Donors and MitoPerSulf. (A) Mechanism of H_2_S release by AP39. The thiocarbonyl group of the 1,2-dithiole-3-thione hydrolyzes to form the corresponding 1,2-dithiole-3-one (RT01) and release H_2_S. RT01 undergoes further hydrolysis to release H_2_S and generate unknown products. (B) Mechanism of H_2_S release by AP123. The thiocarbonyl group of the thiobenzamide hydrolyzes to form the corresponding amide and release H_2_S. (C) Synthesis of MitoPerSulf. (D) Mitochondria-selective H_2_S generation by MitoPerSulf. The mitochondria-targeting triphenylphosphonium group (TPP) leads to uptake of MitoPerSulf into mitochondria where the benzoyl thioester is cleaved by reaction with thiols to generate the unstable persulfide, MitoNAP-SSH that forms persulfides with mitochondrial thiols. These persulfides will then rapidly generate H_2_S and disulfides by reaction with other thiols. For simplicity, reactions with mitochondrial protein thiols are omitted and only reactions with GSH are shown.

The mitochondria-targeted H_2_S donors AP39 and AP123 have also been developed [6,[40], [41], [42], [43]] (Fig. 1A-B). These compounds comprise the mitochondria-targeting lipophilic triphenylphosphonium (TPP) cation [44] coupled via a ten-carbon aliphatic linker to either an anethole dithiolethione moiety in AP39 [41,42] (Fig. 1A), or an hydroxythiobenzamide moiety for AP123 [45] (Fig. 1B). These AP39 and AP123 moieties spontaneously hydrolyse to release H_2_S [[46], [47], [48], [49], [50]]. Furthermore, the initial AP39 hydrolysis product RT01 hydrolyzes further to generate more H_2_S [43] (Fig. 1A). Due to the TPP component these molecules are rapidly concentrated several hundred-fold within mitochondria potentially leading to the local generation of H_2_S. These data were interpreted to suggest that the protective effects against IR injury of AP39, AP123 and RT01 are due to H_2_S release within mitochondria. However, to be effective, mitochondria-targeted H_2_S donors have to be taken up and deliver H_2_S rapidly and selectively within mitochondria during the first few minutes of reperfusion to counteract the oxidative damage caused by the burst of superoxide that occurs at the onset of reperfusion [24,25]. Thus, the time available clinically to reperfuse the ischemic tissue to treat heart attack or stroke is short. As rapid release of H_2_S *in vivo* within this timeframe was never confirmed [51], any acute protective effects of AP39 and AP123 against IR injury may be unrelated to H_2_S release.

Therefore, here we set out to develop a mitochondria-targeted agent that rapidly and selectively released H_2_S solely within mitochondria and could thus be administered upon reperfusion to prevent IR injury. Here we describe the development, assessment and mechanism of action of MitoPerSulf, a mitochondria-targeted molecule that rapidly releases H_2_S within mitochondria *in vivo* and is protective against cardiac IR injury when administered at reperfusion.

## 2 Results

### 2.1 Design and synthesis of the rapid H_2_S releasing agent MitoPerSulf

To generate a molecule that rapidly and selectively releases H_2_S within mitochondria, we exploited the mitochondrial membrane potential-dependent accumulation of TPP cations, the chemistry of persulfides and the high mitochondrial concentration of protein and glutathione (GSH) thiols, which are particularly reactive due to the elevated matrix pH [52]. A mitochondria-targeted persulfide should react rapidly with intramitochondrial thiols to generate persulfides that react further with thiols to generate H_2_S and disulfides [10]. Due to its instability, we protected the persulfide by synthesizing it as a stable thioester with a benzoyl group, that will be rapidly removed by reacting with thiols within mitochondria. The rapid deprotection of the persulfide *in vivo* is essential for the timely generation of H_2_S. The persulfide benzoyl thioester enables this because the low pKa of the persulfide (∼5.45) [53] makes it a good leaving group [54,55], as has been demonstrated previously [10]. To ensure rapid deprotection of the persulfide by thiol attack at the thioester carbonyl, rather than at the α-sulfur atom to form thiobenzoate and a mixed disulfide, we chose a penicillamine-based substituted tertiary persulfide that is sterically constrained at the α-sulfur atom [10]. By conjugating this moiety to a TPP cation via a five-carbon aliphatic linker we constructed a mitochondria-targeted penicillamine-based protected persulfide, MitoPerSulf (Fig. 1C). The synthesis of MitoPerSulf involved modifying MitoNAP-SH, a late-stage intermediate used in the synthesis of MitoSNO [56] by converting it to a mixed disulfide with 2,2ʹ-dithiobis(benzothiazole) and then displacing the 2-mercaptobenzothiazole with thiobenzoic acid [10] (Fig. 1C). It is anticipated that the benzoyl thioester would first be rapidly cleaved by thiols within mitochondria, thus generating the unstable persulfide MitoNAP-SSH that should then transiently persulfidate mitochondrial thiols which then react further with other thiols to release H_2_S (Fig. 1D).

### 2.2 Activation of MitoPerSulf by glutathione *in vitro*

**Fig. 2:**
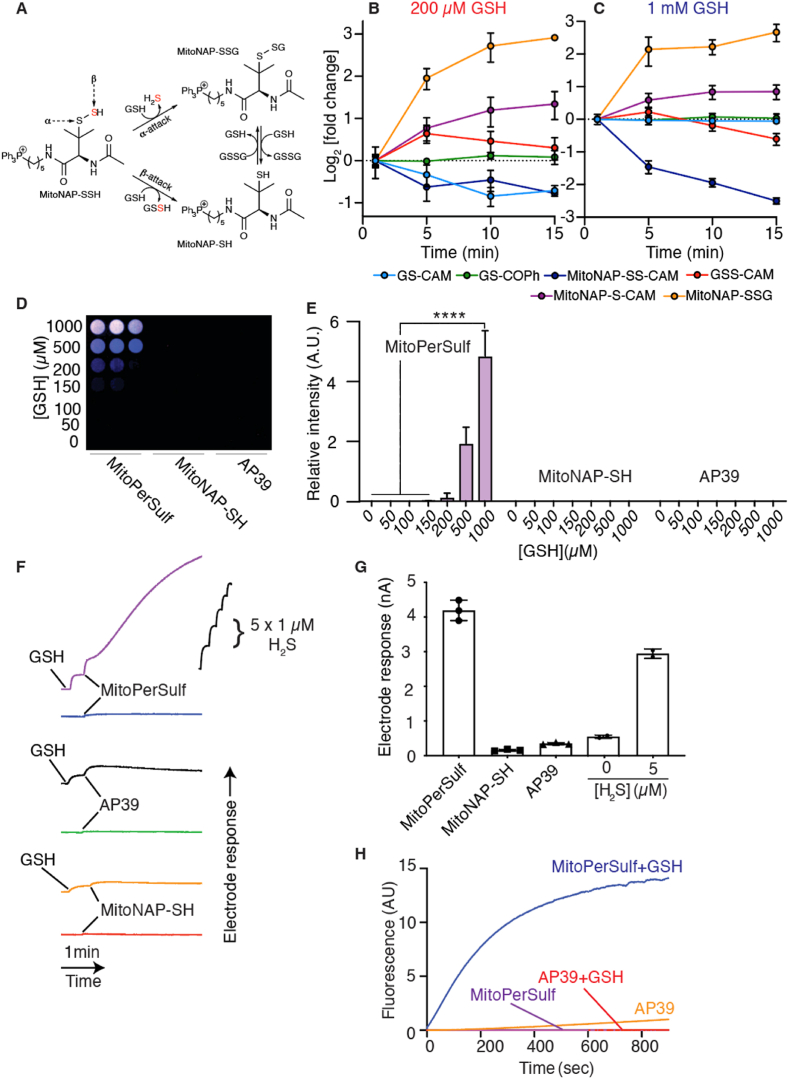
Characterization of Reaction Products of MitoPerSulf and GSH. (A) Schematic of the reaction of GSH with MitoNAP-SSH at the α− or β−sulfur. (B, C) Time course of all intermediary species analyzed in Fig. S2. Data are represented as log_2_ of the fold-change relative to the level at t = 1 min for reaction with 200 μM (B) or 1 mM GSH (C). (D, E) Lead acetate detection of free H_2_S production from reaction of MitoPerSulf (100 μM) and various concentration of GSH in 25 mM HEPES buffer (pH 7.4) at 37 °C. (D) shows a typical result using a false colour scale. (E) Shows the relative intensity (mean ± s.e.m., n = 3, ****p < 0.001, one-way ANOVA). (F, G) Detection of free H_2_S production from reaction of MitoPerSulf, MitoNAP-SH or AP39 (70 μM each) and excess GSH (700 μM) in 25 mM HEPES buffer (pH 7.4) at 23 °C performed using H_2_S-selective micro electrode. (F) Representative traces of electrode response against time. (G) Calibrating the electrode response using anaerobically prepared Na_2_S enabled the [H_2_S] produced after 30 min to be determined. All experiments were performed in triplicates and results are represented as the mean ± s.e.m., n = 3 (****p < 0.001, one-way ANOVA). (H) Fluorescent detection of H_2_S using WSP-5. MitoPerSulf or AP39 (20 μM) were incubated in 25 mM HEPES buffer (pH 7.4) with WSP-5 (10 μM). Where indicated 200 μM GSH was added. The traces are from a typical experiment performed in triplicate. (For interpretation of the references to colour in this figure legend, the reader is referred to the Web version of this article.)

As GSH is the most abundant small molecule thiol within mitochondria, we assessed the activation of MitoPerSulf *in vitro* by reacting it with a 2-fold excess of GSH. This should be sufficient to activate MitoPerSulf, while still allowing MitoNAP-SSH to persist for analysis (Fig. 2, S2). We also used a 10-fold excess of GSH to better mimic the thiol concentration within mitochondria *in vivo* [57] (Fig. 2, S2). To trap the unstable thiol intermediates such as MitoNAP-SSH, we quenched the reaction with excess iodoacetamide (IAM) [58,59]*,* followed by LC-MS/MS analysis to detect the carbamidomethylated (CAM) thiol adducts and other reaction products (Fig. S1). This analysis revealed the rapid formation of a benzoyl thioester of GSH that was complete within 1 min (GSCOPh; Fig. S2A). We also detected the uncapped persulfide MitoNAP-SSH as MitoNAP-SS-CAM, which was rapidly formed within 1 min and subsequently declined over time (Fig. S2B). These findings are consistent with the rapid activation of MitoPerSulf by thiols cleaving the benzoyl thioester to generate MitoNAP-SSH (Fig. 1D). Once formed, reaction of MitoNAP-SSH with other thiols (in this case GSH) could in principle occur at the α-sulfur to generate the disulfide MitoNAP-SSG with H_2_S release, or at the β-sulfur to generate MitoNAP-SH and glutathione persulfide (GSSH) (Fig. 2A). Formation of GSSH, detected as the GSS-CAM adduct, was rapidly generated in the presence of GSH and then declined over time (Fig. S2D), consistent with the initial formation of GSSH from MitoNAP-SSH that subsequently reacts with GSH to generate GSSG and H_2_S (Fig. 1D). The MitoNAP-SSG adduct also increased, albeit more slowly, over time (Fig. S2C), consistent with the subsequent disulfide exchange of MitoNAP-SH and GSSG. We also observed a slight increase in the MitoNAP-S-CAM adduct over time (Fig. S2E), while the GS-CAM adduct only decreased at the lower GSH concentration (Fig. S2F). The lag in formation of IAM adducts of GSSH relative to those of MitoNAP-SSH (Fig. S2G), upon reaction of MitoPerSulf with GSH are consistent with the early formation of MitoNAP-SSH, followed later by the formation of GSSH. Incubation of MitoPerSulf, with a 2-fold excess of GSH generated a little of the GSS-CAM adduct over time, measured as the GSS-CAM/GS-CAM ratio (Fig. S2H), but with a 10-fold excess of GSH there was no increase in GSS-CAM over time, consistent with the rapid reaction of GSSH with thiols. Only GS-CAM, and MitoNAP-S-CAM were observed when MitoNAP-SH was incubated with different concentrations of GSH (data not shown). The relative changes in all these species over time are shown in Fig. 2B and C. Together these data indicate that steric hindrance of the methyl groups prevents GSH reaction at the α-sulfur of MitoNAP-SSH, and that the main pathway is via attack of GSH on the β-sulfur (Fig. 2A) [10].

Our hypothesis was that MitoNAP-SSH should react with thiols to generate free H_2_S. This was confirmed by assessing H_2_S diffusion through air to a lead acetate impregnated filter paper to form lead sulfide (Fig. 2D and E). In contrast, the production of H_2_S by AP39, even in the presence of GSH, was negligible over this time scale (Fig. 2D and E). Generation of H_2_S by MitoPerSulf in the presence of GSH was further demonstrated using an H_2_S electrode (Fig. 2F and G). Again, the production of H_2_S by AP39 over this time scale was negligible, even in the presence of GSH (Fig. 2F and G), consistent with its proposed mechanism as a slow-release H_2_S donor activated by hydrolysis [60]. Finally, we used the fluorescent probe WSP-5, in which a disulfide undergoes nucleophilic attack by HS^−^ followed by cyclization to a fluorescent product [61]. Neither MitoPerSulf nor AP39 showed initial generation of H_2_S, but upon addition of GSH MitoPerSulf rapidly generated H_2_S, while AP39 did not (Fig. 2H).

**Fig. 3:**
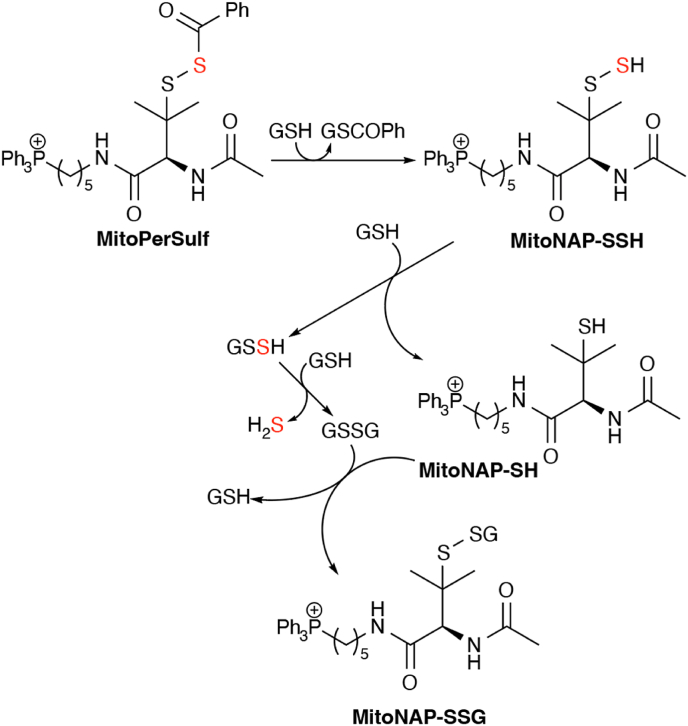
Reaction Mechanism of Thiol-dependent H_2_S Release by MitoPerSulf. MitoPerSulf reacts with thiols (illustrated here solely with GSH) to rapidly form the persulfide, MitoNAP-SSH and GSCOPh. MitoNAP-SSH is further transformed by reacting with GSH to form GSSH and MitoNAP-SH. In the presence of excess GSH, GSSH forms H_2_S *via* formation of GSSG which can react with MitoNAP-SH to form the MitoNAP-SSG.

The proposed reaction scheme for MitoPerSulf with thiols, illustrated using GSH, is shown (Fig. 3). In summary, the spontaneous production of H_2_S by MitoPerSulf and AP39 is very low, but in the presence of excess thiols, as occurs *in vivo*, MitoPerSulf rapidly generates H_2_S, while AP39 does not.

### 2.3 MitoPerSulf is taken up by mitochondria and cells rapidly forming H_2_S

**Fig. 4:**
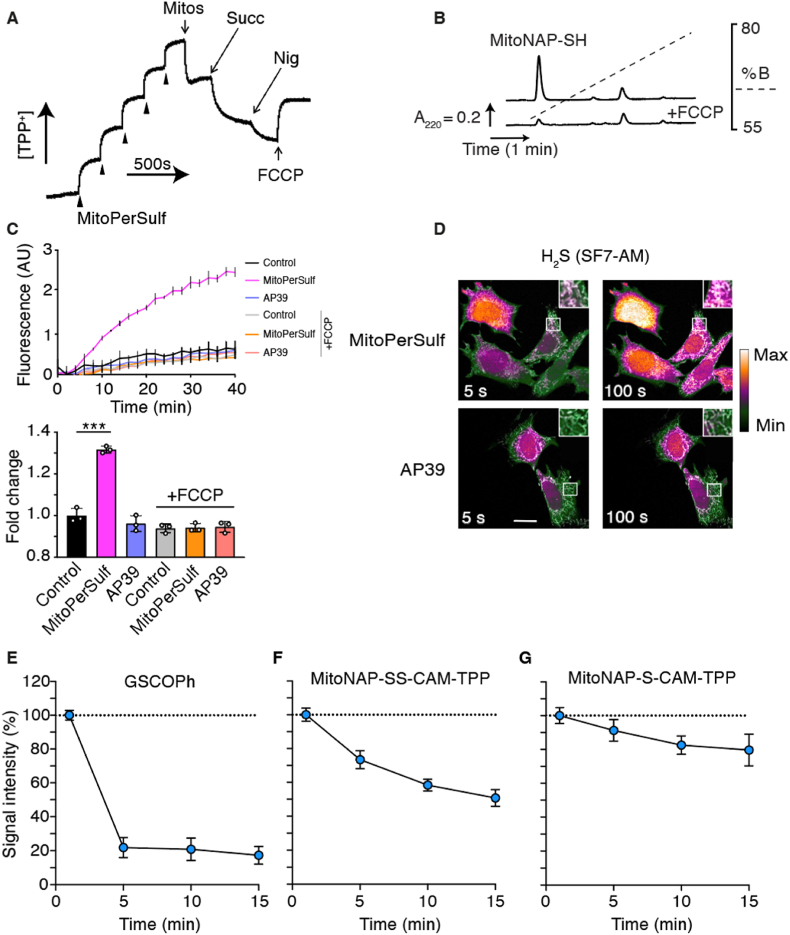
Accumulation and Metabolism of MitoPerSulf in Mitochondria and Cells. (A) Accumulation of MitoPerSulf by energized mitochondria. MitoPerSulf was assessed using a TPP-selective electrode, calibrated by additions of 5 × 1 μM MitoPerSulf followed by rat liver mitochondria (Mitos) (1 mg protein/ml) in the presence of rotenone (4 μg/ml), and then by succinate (Succ; 10 mM), followed by nigericin (Nig; 0.5 μM) and FCCP (0.5 μM) as indicated. (B) Uptake of MitoPerSulf into mitochondria analyzed by RP-HPLC. Mitochondria were incubated with succinate and rotenone as in (A) and MitoPerSulf (5 μM) ± FCCP (0.5 μM) in KCl buffer (pH 7.4) for 3 min at 37°C. Panels (A) and B) are typical results of experiments performed in triplicate. Peak identities were confirmed by use of MitoNAP-SH and MitoPerSulf standards. (C) Effect of MitoPerSulf, and AP39 on H_2_S generation within isolated mitochondria. Rat heart mitochondria (0.5 mg protein/ml) were incubated with 20 μM MitoPerSulf, AP39 or EtOH (vehicle) in KCl buffer as above in the presence of WSP-5 (20 μM) ± FCCP (0.5 μM). The upper panel shows the development of WSP-5 fluorescence over time using a fluorescent plate reader. The lower bar chart shows the fold change of the fluorescence signal at 20 min compared to control. Data are mean ± s.e.m. (n = 3) (***p < 0.001 by Student's *t*-test). (D) Representative confocal microscopy live cell imaging of mitochondrial H_2_S formation by MitoPerSulf using the SF7-AM probe. Mouse embryonic fibroblasts stably expressing the mitochondrial matrix-targeted derivative of the red fluorescent protein (mScarlet) were stained with SF7-AM (2.5 μM) for 40 min, washed and placed in imaging medium. After obtaining the base line signal the indicated compounds were added (20 μM) and imaging was continued. Representative images are shown at indicative time points. Heat map-based green to white H_2_S fluorescence (SF7-AM; *λ*_em_ = 526 nm emission) and mitochondrial fluorescence (grey; mScarlet *λ*_em_ 647 nm), are overlaid. Co-localized signal of the formation of H_2_S in functional mitochondria is highlighted in zoom insets (n = 3, scale bar = 20 μm). (E, F, G) MitoPerSulf metabolism within mitochondria. RHM (1 mg protein/mL) were incubated ± MitoPerSulf (10 μM) in KCl buffer at 37°C supplemented with succinate (10 mM) and rotenone (4 μg/ml). Aliquots were centrifuged (1 min at 17 000×*g*) at the indicated times and precipitated mitochondrial pellets were rapidly resuspended in 50 μL of 40 mM TPP-IAM (200 mM stock solution in MeOH or DMSO) in 100 mM HEPES buffer (pH 7.8). Samples were vortexed and sonicated in a sonic bath at RT in dark for 20 min. Next, 200 μL of ACN were added and samples were placed at −20 °C for 5 min. Samples were centrifuged (10 min at 17 000×g) to pellet proteins and 100 μL of supernatant were retrieved and combined with 400 μl of MS-grade H_2_O containing FA (0.1%). Subsequently, samples were diluted (in 20 % ACN 0.1% FA) as required and analyzed by LC-MS/MS to to assess levels of GSCOPh (E), MitoNAP-SS-CAM-TPP (F) and MitoNAP-S-CAM-TPP (G). Data are mean ± s.e.m. (n = 3). (For interpretation of the references to colour in this figure legend, the reader is referred to the Web version of this article.)

To be an effective mitochondrial H_2_S-generating agent, MitoPerSulf has to be accumulated by mitochondria in response to the membrane potential (Δψ). Using a TPP-selective electrode we showed that MitoPerSulf was accumulated by energized mitochondria and that the dissipation of Δψ with the uncoupler FCCP released the TPP-containing moiety (MitoNAP-SH) from the mitochondria (Fig. 4A). The Δψ-dependent uptake of MitoPerSulf by mitochondria was further confirmed by RP-HPLC analysis of mitochondria pelleted after incubation with MitoPerSulf (Fig. 4B). Only MitoNAP-SH was detected by HPLC following incubation of energized mitochondria with MitoPerSulf, consistent with reduction of MitoPerSulf to MitoNAP-SH by thiols within mitochondria (Fig. 4B). The effect of MitoPerSulf on respiration of isolated mitochondria showed that at high concentrations MitoPerSulf inhibited respiration, while the same concentration of MitoNAP-SH did not (Fig. S3A), suggesting that the effect of MitoPerSulf on respiration was most likely due to the generation of H_2_S, as is explored in detail later.

To investigate the generation of H_2_S within mitochondria, we next measured H_2_S release by MitoPerSulf when incubated with mitochondria in the presence of the fluorescent H_2_S sensor WSP-5 (Fig. 4C). This showed that when succinate was added to drive MitoPerSulf accumulation within mitochondria H_2_S production rapidly increased, but that addition of FCCP to prevent MitoPerSulf uptake blocked H_2_S generation. In contrast, AP39 did not generate H_2_S within mitochondria over this time scale. To examine whether MitoPerSulf can induce the formation of H_2_S within cells, we stably transfected mouse embryonic fibroblasts with a mitochondria-targeted version of the red fluorescent protein mScarlet and used the fluorescent H_2_S sensor SF7-AM, that tends to distribute evenly throughout the cell [62] (Fig. S3B). This showed the rapid and time-dependent formation of H_2_S from MitoPerSulf (Fig. S3B), but limited formation from AP39 over this time scale (Fig. 4D). Colocalization of the SF7-AM and mitochondrial matrix-targeted mScarlet signals showed that the H_2_S signal from MitoPerSulf was present in mitochondria (Fig. 4D, inset), but also diffused throughout the cell (Fig. 4D). Together these data are consistent with rapid accumulation of MitoPerSulf within mitochondria where it generates H_2_S some of which may diffuse out to the rest of the cell.

### 2.4 MitoPerSulf metabolism within mitochondria

To analyze the interaction of MitoPerSulf with mitochondrial thiols we incubated isolated mitochondria with MitoPerSulf and then analyzed extracts by LC-MS/MS. This demonstrated the initial formation of the benzoylated GSH, GSCOPh, which then rapidly decreased (Fig. 4E). In order to increase the sensitivity of the LC-MS/MS detection for the lower amounts of MitoPerSulf metabolites being analyzed, we replaced IAM as the quenching reagent with IAM-TPP [63], an IAM derivative modified to incorporate a TPP cation. Trapping these species as ***X***-CAM-TPP derivatives will introduce a fixed positive charge via the TPP moiety greatly enhancing detection sensitivity by MS (Fig. S1). Using this strategy, we demonstrated the initial formation of MitoNAP-SSH (detected as MitoNAP-SS-CAM-TPP) (Fig. 4F) and MitoNAP-SH (detected as MitoNAP-S-CAM-TPP) (Fig. 4G) within mitochondria. We also attempted to use IAM-TPP to detect GSSH (detected as GSS-CAM-TPP) within mitochondria incubated with MitoPerSulf, but the amounts detected were not significantly above baseline, consistent with the rapid metabolism of GSSH to H_2_S.

MitoNAP-SSH may also directly persulfidate protein thiols. To assess this possibility, we used recombinant Cofilin-1 protein *in vitro*, which contains 4 Cys residues (Fig. S4A), and is known to be persulfidated under certain conditions within cells [64]. We incubated Cofilin-1 with MitoPerSulf and GSH to generate MitoNAP-SSH and then assessed protein persulfidation by trapping with IAM, followed by trypsin digestion and LC-MS analysis to detect the persulfidated peptides (Fig. S4B). We detected two persulfidated peptides at Cys residues C39 and C139 in response to MitoPerSulf (Figs. S4C and D). We were not able to reliably detect persulfidation of cysteine residues C80 and C147. By comparing the relative amounts of the persulfidated Cys residues with those that were free to react with IAM we could estimate the extent of persulfidation as between 10 and 20% under these conditions (Figs. S4C and D). This suggests that MitoPerSulf can potentially lead to protein persulfidation. To assess if MitoPerSulf could lead to protein persulfidation within mitochondria, we incubated heart mitochondria with MitoPerSulf under the same conditions as in Fig. 4 and then analyzed for protein persulfidation using a fluorescence tag switch method [38] followed by analysis of incorporated fluorescence after separation of proteins by SDS-PAGE. However, we did not find consistent increases in fluorescent labelling of individual protein bands on the gels above control (Fig. S5). Furthermore, the negligible amounts of GSSH found when MitoPerSulf was incubated *in vitro* with excess GSH (Fig. 2E) and the lack of detection of GSSH within mitochondria incubated with MitoPerSulf make it likely that the majority of protein persulfides formed by MitoPerSulf are transient and react further to generate H_2_S. Together these data are consistent with the rapid but transient formation of persulfides from MitoPerSulf within mitochondria that rapidly react further with thiols to form H_2_S.

### 2.5 Distribution and cardioprotective effects of MitoPerSulf on acute IR injury *in vivo*

We next used an *in vivo* mouse model of cardiac IR injury to investigate the potential protective effects of MitoPerSulf. First, we analyzed the cardiac uptake of MitoPerSulf *in vivo* in mice following a bolus, intravenous tail vein injection of MitoPerSulf (0.2 mg/kg) with the tissue distribution analyzed by LC-MS/MS spectrometry. Tissues were reduced by addition of dithiothreitol (DTT) during extraction to convert any residual MitoPerSulf derivatives to MitoNAP-SH, thus data are reported as MitoNAP-SH content. As expected from similar TPP-based compounds [65], MitoPerSulf and any derivatives formed over this time scale were rapidly cleared from the plasma (Fig. S6A), leading to their rapid accumulation in the heart (Fig. S6B) as well as into the kidney and liver, with less penetration into the brain, followed by their gradual clearance from these tissues over time (Figs. S6B and C). Therefore, MitoPerSulf is taken up rapidly into the heart (Fig. S6B) following i.v. injection, making it suitable as a potential protective agent against cardiac IR injury for administration upon reperfusion.

**Fig. 5:**
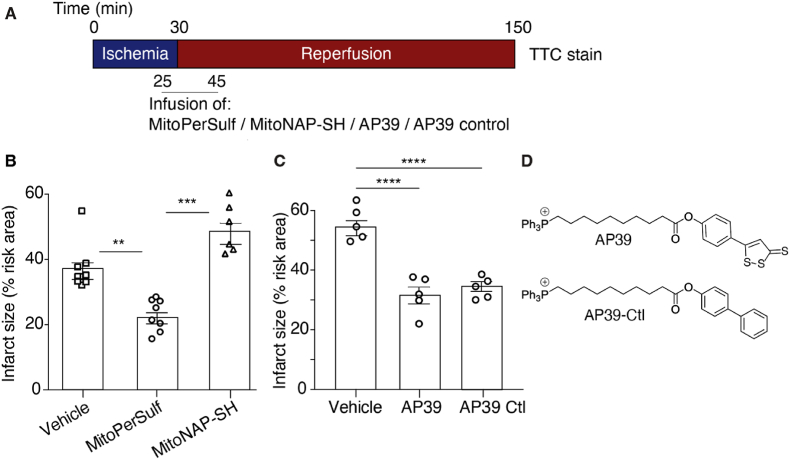
Cardioprotective effect of MitoPerSulf on cardiac IR. (A) Schematic of cardiac IR injury experiments. Mice were subjected to 30 min ischemia by ligation of the left anterior descending coronary artery (LAD) ligation followed by 120 min of reperfusion. Compounds (10 μg/kg) or vehicle (0.5% DMSO) were infused into the tail vein at 5 μL/min for 20 min starting 10 min before reperfusion. Myocardial infarct size was then determined as a percentage of the area at risk quantified from a single mouse by triphenyl tetrazolium chloride (TTC) stain. (B, C). Effect of compounds on infarct size. (B) MitoPerSulf or MitoNAP-SH. (C) AP39 or AP39 control compound. *p < 0.05, **p < 0.01, one-way ANOVA, n = 5–8 ± s.e.m. (D) Structures of AP39 and the AP39 control compounds.

Next, we assessed the protective effects of MitoPerSulf against cardiac IR injury by performing left anterior descending (LAD) coronary artery ligation in mice, followed by reperfusion and assessment of infarct size (Fig. 5A). Infusion of MitoPerSulf for 20 min starting 5 min before reperfusion resulted in a dose-dependent reduction of infarct size that reached a maximum at 10 μg/kg/min (Fig. S6D). Comparison of the most effective dose with the same concentration of MitoNAP-SH showed that MitoPerSulf was protective while MitoNAP-SH was not (Fig. 5B). As MitoNAP-SH is structurally very similar to MitoPerSulf and it is produced upon metabolism of MitoPerSulf within mitochondria this suggests that the protection against cardiac IR injury by MitoPerSulf is due to its rapid generation of H_2_S within mitochondria. Furthermore, as the uptake of MitoNAP-SH into mitochondria *in vivo* will be to a very similar extent as for MitoPerSulf, the protective effects of MitoPerSulf are not due to the disruption of mitochondrial function by the alkylTPP molecule.

Acute protection against cardiac IR injury has been reported when AP39 is administered upon reperfusion [40,41,66]. We confirmed this protection here (Fig. 5C). The tacit assumption in these earlier publications was that the mode of action of AP39 was through H_2_S release *in vivo,* but this was not demonstrated. AP39 releases H_2_S far more slowly than MitoPerSulf within mitochondria (Fig. 4) making it unlikely that the protection against acute cardiac IR injury by AP39 is due to rapid H_2_S release and may instead be due to off-target effects. To explore this possibility, we made a chemically similar control version of AP39 that does not release H_2_S (Fig. 5D). AP39's reactive group is comprised of two planar highly conjugated rings capable of conjugation to each other at the oxygen atom of the ester. These rings are linked by a rotatable bond allowing other conformations (Fig. S7A). The planar 1,2-dithio-3-thione is weakly aromatic [67,68] and carbon and sulfur have very similar electronegativities. Therefore, we reasoned that a planar aromatic phenyl ring with the same number of heavy atoms would mimic its size, shape, and overall lipophilicity well (Fig. S7A). To confirm this the logP was calculated for the reactive head group of AP39 and the corresponding phenyl analogue using a consensus model built on Chemaxon and Klopman et al. [69] models using the PHYSPROP database (Fig. S7A). Calculating only the head group simplifies the calculation and avoids complications associated with the modelling of logPs of single ions [70,71]. The similarity of the logPs calculated for the head groups gave confidence that a control with the same TPP targeting group and alkyl linker would have similar physicochemical properties and thus uptake into mitochondria *in vivo* (Fig. S7B). RP-HPLC confirmed this similarity (Fig. S7C). The AP39 control compound was indeed as protective against cardiac IR injury as AP39 in the LAD model (Fig. 5C), further confirming that the protection afforded by AP39 is not due to the release of H_2_S, but to off-target effects, which may be due to accumulation of the hydrophobic alkylTPP molecule within mitochondria affecting organelle function. Of course, the slow release of H_2_S by AP39 may protect against tissue damage that occurs in the hours following reperfusion, but this was not explored here. In contrast, the protection against cardiac IR injury by MitoPerSulf, which rapidly releases H_2_S, but not by the chemically closely related compound MitoNAP-SH which does not release H_2_S, suggests that the rapid release of H_2_S within mitochondria in the heart is protective against IR injury.

### 2.6 Mechanism of protection by MitoPerSulf against acute cardiac IR injury

**Fig. 6:**
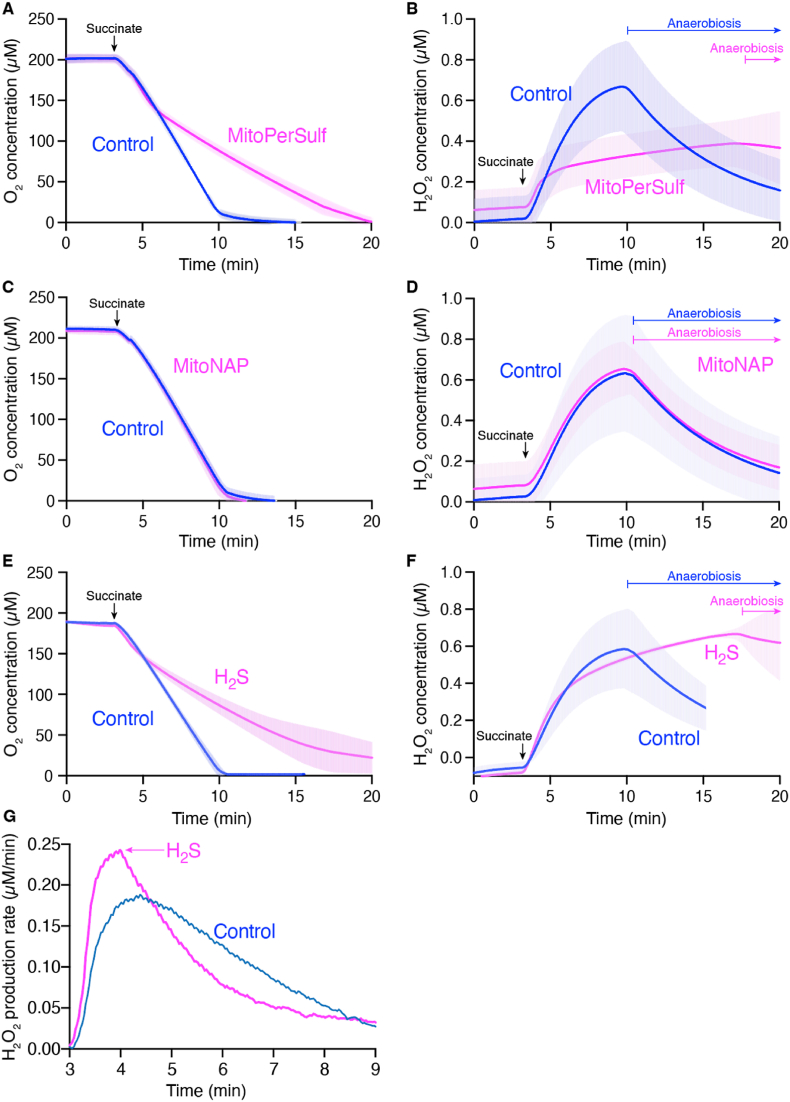
Effect of MitoPerSulf on mitochondrial respiration and superoxide production by RET *in vitro*. Rat heart mitochondria RHM (1 mg protein/2 mL) were resuspended in KCl buffer in an Oroboros Oxygraph-2k system, and respiration and H_2_O_2_ generation by RET were initiated by adding 10 mM succinate followed 1 min later by vehicle, or 10 μM MitoPerSulf, MitoNAP or Na_2_S. Oxygen consumption (A, C, E) and H_2_O_2_ formation by RET (B, D, F) were recorded. The effect of vehicle on these was compared with that of 10 μM MitoPerSulf (A, B), MitoNAP (C, D) or Na_2_S (E, F). The first derivative of the data in panel F are replotted in panel G. Data are represented as means ± s.e.m. (shading) of n = 3 of one mitochondrial preparation, which was repeated with the same outcome on 4 independent mitochondrial preparations.

We next explored the mechanism of protection against cardiac IR injury by the rapid burst of H_2_S generation produced by MitoPerSulf within mitochondria. Mitochondrial superoxide production by reverse electron transport (RET) upon reperfusion is thought to initiate the damaging cycle that leads to tissue damage [24,25]. To explore whether H_2_S could alter this process, we investigated the effect of MitoPerSulf on superoxide production by RET in isolated mitochondria (Fig. 6). Addition of MitoPerSulf decreased respiration compared to control and this inhibitory effect of MitoPerSulf increased as the oxygen concentration diminished, thereby extending the time taken to remove all the oxygen from the incubation (Fig. 6A). In parallel, we measured the extent of superoxide production by RET through the generation of H_2_O_2_. In control mitochondria there was considerable H_2_O_2_ generation that slowed as the oxygen level fell (Fig. 6B). Following anaerobiosis the fluorescence due to Resorufin decreased, due to its enzymatic reduction to dihydroresorufin upon anaerobic conditions [72], that is likely to disrupted by the presence of H_2_S [73]. In contrast, addition of MitoPerSulf greatly decreased H_2_O_2_ generation, in parallel with its effect of on respiration (Fig. 6A). The control compound MitoNAP-SH had no effect on respiration (Fig. 6C), or on the generation of H_2_O_2_ (Fig. 6D). Thus, the effect of MitoPerSulf on respiration and on the generation of H_2_O_2_ is not due to any non-specific effects of the accumulation of the TPP cation on the mitochondria but instead is due to the generation of H_2_S within mitochondria. To investigate this further, we incubated mitochondria in the presence of H_2_S by adding Na_2_S, which had a very similar effect on mitochondrial respiration (Fig. 6E). The addition of H_2_S also slowed the rate of H_2_O_2_ generation (Fig. 6F), compared to the control incubation. To better illustrate the effect of adding H_2_S on H_2_O_2_ generation, we plotted the slope of the data in Fig. 6F against time, which showed that the rate of H_2_O_2_ generation decreased immediately upon addition of H_2_S (Fig. 6G), while in contrast in the control incubation the rate of H_2_O_2_ generation decreased gradually as the O_2_ concentration decreased. These data suggest that the generation of H_2_S from MitoPerSulf within mitochondria disrupts respiration and thereby prevents mitochondrial superoxide production by RET.

## 3 Conclusions

The role of H_2_S donors as potential therapies has attracted considerable interest. In particular, it has been proposed that these donors could be used to prevent the damage associated with IR injury in heart attack and stroke by selective targeting to mitochondria. However, for the clinical treatment of IR injury it is necessary to add the protective agent upon reperfusion. While the targeting of compounds to mitochondria by conjugation to the lipophilic TPP cation is well established [44], the mitochondria-targeted H_2_S donors developed to date such as AP39 release H_2_S slowly, suggesting that any acute protective effects are not due to H_2_S release. Thus, the potential therapeutic utility of acute release of H_2_S within mitochondria remains unexplored. Here we addressed this by developing MitoPerSulf, a mitochondria-targeted H_2_S donor. We used a TPP cation to target MitoPerSulf to mitochondria *in vivo*, following intravenous administration. By adapting persulfide chemistry we were able to mask a reactive persulfide moiety that then rapidly releases H_2_S within mitochondria. This development opens the way for the development of further donors designed to rapidly release H_2_S within mitochondria.

Most importantly, we showed that MitoPerSulf was acutely protective in the *in vivo* LAD model of cardiac IR injury. In doing this, we utilized appropriate control compounds to show that the protective effects of MitoPerSulf were due to rapid H_2_S release and not to off-target effects of the mitochondria targeting TPP moiety. We also demonstrated, through the use of an appropriate control compound, that the reported protective effects of AP39 against IR injury were due to off-target effects resulting from the physicochemical properties of molecules that have a targeting TPP moiety linked by a long alkyl chain to a nonpolar biaryl system. Thus, for the first time we have demonstrated that the acute generation of H_2_S within mitochondria is a viable therapeutic strategy against IR injury.

**Fig. 7:**
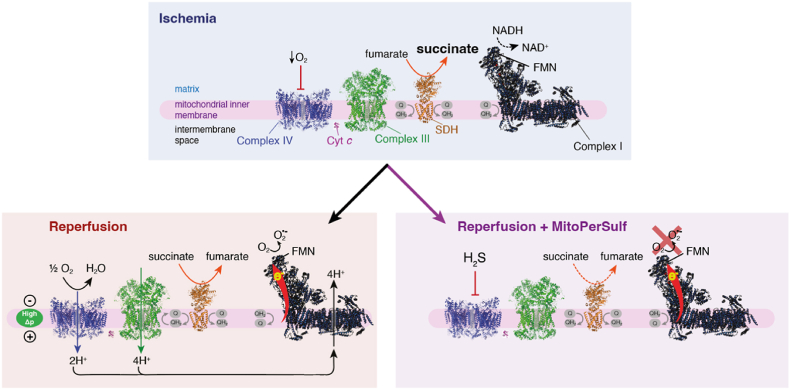
Schematic of effect of rapid H_2_S generation by MitoPerSulf on cardiac IR injury *in vivo.* The upper panel shows the accumulation of succinate during ischemia. The lower left panel shows that this succinate then drives mitochondrial superoxide production by reverse electron transport (RET) upon reperfusion that depends on the development of a high proton motive force (Δp) upon reperfusion. The lower right panel shows that the H_2_S generated by MitoPerSulf reversibly inhibits complex IV/cytochrome *c* oxidase thereby preventing the build-up of Δp upon reperfusion and thus blocking superoxide production by RET.

The mechanism of protection by acute H_2_S generation within mitochondria was also determined. H_2_S is well established to bind selectively and reversibly to cytochrome *c* oxidase and thereby to inhibit mitochondrial respiration. We showed that MitoPerSulf acted in this way by rapidly inhibiting respiration and that its inhibitory potency increased as the oxygen concentration decreased. This is consistent with the well-established competition between O_2_ and H_2_S at cytochrome *c* oxidase. This inhibition of respiration will lower the mitochondrial protonmotive force and should thereby prevent the ability of mitochondrial complex I to generate superoxide by RET. We demonstrated this in isolated mitochondria with both MitoPerSulf and with pure H_2_S. Thus, we suggest that the protective effects of acute generation of H_2_S within mitochondria against IR injury is largely by preventing the burst of superoxide production by complex I upon reperfusion (Fig. 7). Even so, it is important to note that additional protective effects of H_2_S, such as by preventing overoxidation of protein thiols, are not excluded. The reversible inhibition of cytochrome *c* oxidase by H_2_S is similar to that by nitric oxide (NO) [56] and suggests that acute generation of NO within mitochondria may also be protective against IR injury by a similar mechanism. Indeed, in earlier work we developed a mitochondria-targeted NO donor (MitoSNO) which was acutely protective against IR injury [56]. While we interpreted this as being due to the selective *S*-nitrosation of Cys 39 on complex I, thereby preventing RET, the degree of exposure of this Cys residue *in vivo* has been reassessed [63]. Thus, the protection against IR injury by MitoSNO may have been, at least in part, due to the reversible inhibition of cytochrome *c* oxidase decreasing respiration and thereby decreasing mitochondrial superoxide production at complex I upon RET.

In summary, we have developed the first approach to rapidly and selectively generate H_2_S within mitochondria *in vivo*. Using this approach, we were able to demonstrate that H_2_S is acutely protective against IR injury by reversibly inhibiting respiration at cytochrome oxidase and thereby preventing superoxide production at complex I.

## 4 Materials and methods

### 4.1 Animals

All procedures were carried out in accordance with the UK Animals (Scientific Procedures) Act 1986 and the University of Cambridge Animal Welfare Policy. Procedures were approved to be carried out under the Project Licenses: 70/7963, 70/8238. Female Wistar rats, or male or female C57BL/6J mice (both Charles River Laboratories, UK) were maintained in pathogen-free facilities with *ad libitum* chow and water until being 8–20 weeks of age for experimental use.

### 4.2 Chemicals

All buffers used in this study were prepared with salts of highest purity using MiliQ water (18.2 μ Ω), supplemented with Chelex-100 resin. All chemicals were obtained from commercial sources, except TPP-IAM, MitoNAP-SH, MitoPerSulf, AP39 control and (9-carboxynonyl)triphenylphosphonium bromide (Table S1). TPP-IAM and MitoNAP-SH (MitoNAP) were prepared as described previously [56,63]. MitoPerSulf was prepared by converting MitoNAP-SH to a mixed disulfide by reaction with 2,2-dithiobis(benzothiazole) and then displacing the 2-mercaptobenzothiazole with thiobenzoic acid. In brief, AP39 control was prepared by coupling (9-carboxynonyl)triphenylphosphonium bromide, prepared by the method of Thurnhofer et al. [74], to 4-phenylphenol using *N*-(3-dimethylaminopropyl)-*N*′-ethylcarbodiimide hydrochloride (EDCI) and 4-dimethylaminopyridine (DMAP). Synthetic procedures for MitoPerSulf and AP39 control are given below. NMR data are deposited at: https://doi.org/10.5525/gla.researchdata.1304.

#### 4.2.1 5-(2′-Acetylamino-3′-benzoyldisulfanyl-3′-methylbutyrylamino)pent-1-yl]-triphenylphosphonium methanesulfonate MitoPerSulf

MitoNAP mesylate [200 mg, 0.320 mmol, 1 eq., prepared by the method described previously [56], was added to a solution of 2,2′-dithiobis(benzothiazole) (144 mg, 0.432 mmol, 1.35 eq.) in CHCl_3_ (12 mL). The solution was stirred at RT for 48 h then concentrated under vacuum. Automated flash chromatography [SiO_2_, dichloromethane-MeOH (99:1) to (70:30)] then gave the mixed disulfide intermediate as a white solid (165 mg, 65 %). The mixed disulfide (120 mg, 0.146 mmol, 1 eq.) was dissolved in CDCl_3_ (1.9 mL) and thiobenzoic acid (75 μL, 0.59 mmol, 4 eq.) was added. The reaction mixture was stirred at RT for 80 min and the crude mixture was purified by automated flash chromatography [SiO_2_, dichloromethane-MeOH (99:1) increasing to (70:30)] to provide MitoPerSulf as an oil (115 mg, 100 %). *R*_*f*_ [SiO_2_, CH_2_Cl_2_: MeOH (99:1)]: 0.17. *ʋ*_max_ (ATR): 3279 (NH), 2931 (CH), 2870 (CH), 1651 (C=O) cm^−1^. *δ*_H_ (400 MHz, CDCl_3_): 8.16 (1H, t, *J* = 5.4 Hz, CH_2_N*H*), 8.01–7.99 (2H, m, 2 × *ortho*-H of benzoyl group), 7.82–7.66 (16H, m, Ph_3_P and CHN*H*), 7.59 (1H, broad t, *J* = 7.5 Hz, *para*-H of benzoyl group), 7.45 (2H, broad t, *J* = 7.7 Hz, 2 × *meta*-H of benzoyl group), 4.68 (1H, d, *J* = 9.4 Hz, CH), 3.58–3.51 (2H, m, CH_2_P^+^), 3.35–3.19 (2H, m, C*H*_*2*_NH), 2.74 (3H, s, SO_3_CH_3_), 2.12 (3H, s, COCH_3_), 1.74–1.61 (6H, m, 3 × CH_2_), 1.49 (3H, s, CH_3_), 1.42 (3H, s, CH_3_). *δ*_C_ (126 MHz, CDCl_3_): 191.81 (C), 170.69 (C), 169.51 (C), 135.88 (C), 135.16 (d, *J* = 2.8 Hz, CH), 134.09 (CH), 133.63 (d, *J* = 10.0 Hz, CH), 130.60 (d, *J* = 12.6 Hz, CH), 128.91 (CH), 127.96 (CH), 118.46 (d, *J* = 86.3 Hz, C), 60.21 (CH), 54.37 (C), 39.74 (CH_3_), 38.50 (CH_2_), 27.85 (CH_2_), 27.22 (d, *J* = 16.9 Hz, CH_2_), 25.86 (CH_3_), 25.35 (CH_3_), 23.33 (CH_3_), 22.10 (d, *J* = 43.9 Hz, CH_2_), 21.88 (d, *J* = 1.4 Hz, CH_2_). δ_P_ (162 MHz, CDCl_3_): 24.25 (1P, s). LRMS (ESI^+^): 657 [M^+^, 100%], 625 (51), 536 (50), 413 (35). HRMS (ESI^+^): 657.2350. C_37_H_42_N_2_O_3_PS_2_ requires M^+^ 657.2369.

#### 4.2.2 Synthesis of [9-(4′-phenylphenoxycarbonyl)nonyl]triphenylphosphonium chloride

EDCI (63 mg, 0.330 mmol, 1.5 eq.) was added to a solution of (9-carboxynonyl)triphenylphoshonium bromide (113 mg, 0.220 mmol 1.0 eq.), DMAP (2.0 mg, 0.02 mmol, 0.1 eq.) and 4-phenylphenol (56 mg, 0.330 mmol, 1.5 eq.) in dry dichloromethane (2 mL). After stirring overnight at RT under an atmosphere of argon the solution was diluted with dichloromethane and washed with 1 M hydrochloric acid, brine and then NaHCO_3_ solution. The organic layer was dried over magnesium sulfate and concentrated under vacuum. Automated flash chromatography [dichloromethane-MeOH (100:0) to (91:9)] gave the phosphonium salt as a white solid. (102 mg, 75%). *δ*_H_ (400 MHz, CDCl_3_): 7.84–7.72 (9H, m, 6 × *ortho* and 3 × *para* H's of PPh_3_), 7.71–7.62 (6H, m, 6 × *meta* H's of PPh_3_), 7.53 (2H, d, *J* = 8.5 Hz, H-3′ and H-5′), 7.53–7.49 (2H, m, H-2″ and H-6″), 7.43–7.36 (2H, m, H-3″ and H-5″), 7.34–7.27 (1H, m, H-4″), 7.10 (2H, d, *J* = 8.6 Hz, H-2′ and H-6′), 3.78–3.64 (2 H, m, PCH_2_), 2.51 (2H, t, *J* = 7.4 Hz, CH_2_CO_2_), 1.68 (2 H, qn, *J =* 7.4 Hz, C*H*_*2*_CH_2_CO_2_), 1.63–1.53 (4H, m, 2 × CH_2_), 1.40–1.20 (8H, m, 4 × CH_2_). *δ*_C_ (101 MHz, CDCl_3_): 172.43 (C), 150.21 (C), 140.39 (C), 138.85 (C), 135.06 (d, *J* = 3.0 Hz, CH), 133.68 (d, *J* = 9.9 Hz, CH), 130.55 (d, *J* = 12.6 Hz, CH), 128.83 (CH), 128.13 (CH), 127.37 (CH), 127.12 (CH), 121.90 (CH), 118.46 (d, *J* = 85.7 Hz, C), 34.38 (CH_2_), 30.41 (d, *J* = 15.6 Hz, CH_2_), 29.09 (CH_2_), 29.08 (CH_2_), 28.99 (CH_2_), 28.95 (CH_2_), 24.86 (CH_2_), 22.68 (d, *J* = 4.6 Hz, CH_2_), 22.56 (d, *J* = 49.8 Hz, CH_2_). δ_P_ (162 MHz, CDCl_3_) 24.28. HRMS (ESI^+^): 585.2912. C_40_H_42_O_2_P requires M^+^ 585.2917.

### 4.3 RP-HPLC analysis

Reverse phase HPLC (RP-HPLC) on a C18 column (Jupiter 300 Å, Phenomenex) with a Widepore C18 guard column (Phenomenex) and a Gilson 321 pump was used for the separation of MitoPerSulf, MitoNAP-SH and their derivatives. Samples were injected through a 0.22 μm PVDF filter (Millipore) and buffer A (0.1 % trifluoracetic acid (TFA) in water (v/v)) and B (0.1 % TFA/acetonitrile (v/v)) was run with the gradient: 0–2 min, 5 %B; 2–17 min, 5–100 % B; 17–19 min, 100 % B; 19–22, 100-5 % B. Peaks at 220 nm were detected with a Gilson UV/VIS 151 spectrophotometer. MitoPerSulf and MitoNAP-SH stock solution in ethanol were used to identify peak elution times. To assess the stability in aqueous buffer and the reaction with glutathione (GSH), MitoPerSulf was incubated in KCl buffer (120 mM KCl, 10 mM 4-(2-hydroxyethyl)-1-piperazineethanesulfonic acid (HEPES), 1 mM ethylene glycol tetraacetic acid (EGTA), 1 mM MgCl_2_ and 5 mM KH_2_PO_4_, pH 7.4) ± GSH at room temperature in the dark, and then mixed with buffer B and analyzed by RP-HPLC.

### 4.4 Detection of H_2_S by fluorescence sensors

To access the H_2_S formation from MitoPerSulf, the fluorescent-based sensors WSP-5 was used. Initially, the spectral properties of WSP-5 (10 μM) were analyzed in 25 mM HEPES buffer (pH 7.4) by exposing it to various concentration of MitoPerSulf, MitoNAP-SH, AP39 (0–25 μM) or GSH (0–1 mM) using high precision spectrofluorometer equipped with stirring and heating block (Shimadzu). For quantification studies, experiments with various concentration of MitoPerSulf and GSH in 25 mM HEPES buffer (pH 7.4) were performed in glass bottom 96 well plate by using SpectraMAX platereader (Molecular Devices). WSP-5 was excited at 502 nm and emission recorded at 525 nm.

### 4.5 Detection of H_2_S by amperometry

H_2_S release from MitoPerSulf and GSH was accessed by using H_2_S sensitive electrode. 5 mm fibre wire H_2_S microelectrode (WPI) was connected to Apollo 4000 free radical analyzer (WPI) and polarized overnight in 10 mM phosphate buffer saline (Gibco) under the 150 mV until reaching the stabile baseline. Amperometric traces in time were obtained by performing the reaction in 3 mL of 25 mM HEPES (pH 7.8) or 10 mM PBS buffer (pH 7.8) under constant and stable stirring and temperature in a multi-port reaction chamber (WPI). Reaction was performed by injecting the various concentration of GSH (0–1 mM) followed by injecting boluses of different concentrations of MitoPerSulf, MitoNAP-SH or AP39 (0–100 μM) from DMSO-based stock solutions into the reaction chamber. Results were obtained by measuring the difference of maximum signal obtained before and after the injections (p(A)_max_ - p(A)_min_ = Δp(A)) for each experimental condition. The H_2_S electrode was calibrated using 25 mM HEPES buffer (pH 7.8) and anaerobically prepared solutions of anhydrous and ultra-pure Na_2_S (Sigma Aldrich Product. Code. 407410) in Chelex-100 treated and argon-purged MiliQ dH_2_O prepared and used at the same day.

### 4.6 Detection of diffusible H_2_S by the lead acetate assay

Release of hydrogen sulfide in the gas phase was assessed using lead (II) acetate [75]. Lead (II) acetate-impregnated filter paper was prepared by soaking clean sheets of Whatman filter paper (# 3030-917) in 20 mM lead (II) acetate in dH_2_O for 20 min and drying them for 2 h at 50°C. Upon drying, lead acetate impregnated paper was stored protected from light at room temperature in a dry and sealed glass container. In brief, 100 μL of reaction mixture containing 100 μM of MitoPerSulf, MitoNAP-SH or vehicle (EtOH) and different concentrations of GSH ranging from 0 to 1 mM in 25 mM HEPES buffer (pH 7.8) was placed in 96-well plate and covered with lead (II) acetate-impregnated filter paper leaving approximately 5 mm of head space between liquid phase and the filter paper. 96-well plate with samples was incubated at 50°C in the oven for 2 h to allow efficient evaporation and accumulation of H_2_S in the head space of well plate and after the incubation the filter paper containing developed lead (II) sulfide spots was immediately scanned using bio scanner (HP) and analyzed by densitometry (ImageJ).

### 4.7 Generation of lentiviral particles and transduction of MEFs

MTS-Scarlet was amplified by PCR with specific oligonucleotides using pMTS_mScarlet_N1(Addgene; #85057) plasmid. This insert was introduced into the pWPXLd-IRES-HygroR lentiviral expression vector, modified versions of pWPXLd (Addgene; #12258), by restriction enzyme digestion with PmeI and BamHI and ligation with T4 DNA ligase (New England Biolabs). Lentiviral particles were generated in HEK293T packaging cells by co-transfection of the lentiviral expression vector with the packaging psPAX2 (Addgene; # 12260) and envelope pMD2.G (Addgene; # 12259) vectors with FuGENE HD (Promega) according to manufacturer's instructions. Mouse embryonic fibroblast cells (MEFs) were transduced with previously generated lentiviral particles with Polybrene (Merck, TR-1003) for 24 h. Transduced cells were then selected for resistance using hygromycin B (Roche, 10843555001) at 50 μg/mL.

### 4.8 Detection of H_2_S by fluorescent microscopy

MEFs stably expressing the fluorescent mitochondrial matrix red protein, MTS-mScarlet were grown in high glucose glutaMAX containing DMEM medium supplemented with 10 % FBS, 1 % Streptomycin-Penicillin solution and at 37°C under the atmosphere of 5 % CO_2_. Upon reaching the 80 % confluency, cells were detached using 0.25 % trypsin and plated in glass bottom 35-mm high μ-Dish (ibidi, Germany) at 3 × 10^4^ cells per dish. After attachment cells were stained with 2.5 μM SF7-AM in complete cell medium for 40 min in dark at 37 °C under the atmosphere of 5 % CO_2_. After staining, cells were washed three times with phenol red-free full DMEM and mounted on the microscope stage. For some experiments cells were washed and imaged using phosphate buffer saline (PBS). 180 images per sample were obtained during the 900 s of live cell imaging (integration time: 5 s) at 37^o^C and stimulation of H_2_S production was initiated by adding the boluses of 20 μM MitoPerSulf or AP39 directly into dishes 10 s upon starting the time-lapse video recording. Fluorescence values were collected every 5 s for 15 min. Images were acquired using a 100x objective of the Nikon Eclipse Ti-E microscope, coupled to an Andor Dragonfly spinning disk confocal system equipped with an Andor Ixon camera, and 488 nm and 561 nm excitation lasers were used for SF7-AM and MTS-mScarlet, respectively. All images were postprocessed under the same parameters using ImageJ software (NIH) and for enhanced visualisation the original SF7-AM fluorescence was presented using the specific heat map projection of signal (ImageJ).

### 4.9 Mitochondria preparations

Rat liver and heart mitochondria (RLM and RHM respectively) were prepared by homogenization of heart tissue obtained from 10 to 12 weeks old Female Wistar rats (Charles River, UK) that were killed by stunning and cervical dislocation, in STEB buffer (250 mM sucrose, 5 mM Tris-HCl and 1 mM EGTA, pH 7.4). Following homogenization, mitochondria were isolated by differential centrifugation (2 x 2450 × *g* for 5 min, 2 x 9150 × *g* for 10 min at 4°C). STE buffer was supplemented with 0.1% fatty acid–free BSA for isolation of RHM. Protein concentration was determined by the bicinchoninic acid (BCA) assay using BSA as a standard.

### 4.10 Mitochondrial uptake of MitoPerSulf

Mitochondrial uptake of MitoPerSulf was assessed using TPP-selective electrode. The electrode was calibrated with five boluses of 1 μM MitoPerSulf followed by 1 mg/mL of RLM in KCl buffer (120 mM KCl, 10 mM HEPES, 1 mM EGTA, 1 mM MgCl_2_ and 5 mM KH_2_PO_4_, pH 7.4). 5 mM succinate was then added to energize RLM after which, the H^+^/K^+^ ionophore nigericin (0.5 μM) and the uncoupler carbonyl cyanide p-trifluoromethoxy- phenylhydrazone (FCCP, 0.5 μM) were added to maximize and collapse the mitochondrial membrane potential, respectively. The uptake was also analyzed with RP-HPLC analysis. RLM were incubated with 5 mM succinate and 4 μg/mL rotenone and 5 μM MitoPerSulf in KCl buffer ± 0.5 μM FCCP to collapse the membrane potential respectively. Compounds in the mitochondria were extracted from mitochondrial pellet with mixture of 20 % Acetonitrile/0.1 % TFA in water (v/v) after 3 min incubation period and detected by RP-HPLC as described above.

### 4.11 Detection of H_2_S release from mitochondria

Freshly isolated RHM (0.5 mg protein/sample) were resuspended in KCL buffer containing 10 mM succinate, 20 μM WSP-5 and/or 0.5 μM FCCP and aliquoted at 180 μL/well in glass-bottom black 96-well plate (Greiner, USA). Immediately upon distribution in well plate, samples were supplemented with 20 μM of MitoPerSulf, AP39 or EtOH (vehicle), and transferred in to a platereader. WSP-5-based fluorescence was measured at various time points using 502 nm excitation and 525 nm emission wavelength in SpectraMAX plate reader (Molecular Devices) at 37°C. Each experimental condition is performed in triplicate.

### 4.12 Mitochondrial respiration and superoxide production *in vitro*

Oxygen consumption and superoxide production were determined using the high resolution O2k oxygraph (Oroboros Instruments). Freshly isolated RHM (1 mg protein) were resuspended in 2 mL of KCL buffer supplemented with 17.6 U SOD, 8.76 U HRP, 12.5 μM Amplex Red and 3 μM BSA and oxygen consumption and superoxide production were induced by simultaneous addition of 10 mM of succinate in each chamber under the constant stirring and constant temperature (T = 37°C). After 1 min, the indicated compounds and control (EtOH) were added and recording of amperometric and fluorescence changes was continued for 25 min. Obtained results of all measurements are presented as means ± s.e.m. of n = 3, repeated on 4 different occasions.

### 4.13 Pharmacokinetics analysis

200 μg/kg MitoPerSulf in 100 μL of saline was administered by tail-vein injection in Wild-type male C57BL/6 mice. Tissues were collected after respective time periods, frozen in liquid nitrogen and then stored at −80°C. MitoPerSulf and its derivatives inside the tissues were reduced to MitoNAP-SH by addition of 0.3 M DTT during the procedure and then MitoNAP-SH, in homogenate was extracted with 0.1 % TFA/acetonitrile and its amount was analyzed by LC-MS/MS as described.

### 4.14 MS method development for detection of reaction metabolites

The mass spectrometric fragmentation patterns for reaction intermediates/metabolites of MitoPerSulf were determined in samples from *in vitro* kinetic experiments of MitoPerSulf in the presence of GSH. Samples were quenched with IAM or TPP-IAM and prepared as follows. IAM quenched samples were prepared by incubating 100 μM of MitoPerSulf with 1 mM GSH in 25 mM HEPES buffer (pH 7.8) at 37°C for 15 min. Fractions of 50 μL were taken after 1, 5, 10 and 15 min and were immediately mixed with 20 mM iodoacetamide (from 200 mM fresh stock solution in dH_2_O) and incubated at RT in dark for 20 min. TPP-IAM quenched samples were prepared by incubating 100 μM of MitoPerSulf with 1 mM GSH in 25 mM HEPES buffer (pH 7.8) at 37°C for 15 min. Fractions of 50 μL were taken after 1, 5, 10 and 15 min and were immediately mixed with 20 mM TPP-IAM (from 200 mM fresh stock solution in MeOH) and incubated at RT in dark for 20 min. Fragmentation patterns were determined by direct infusion of appropriately diluted samples (in 20% ACN, 0.1 % FA) at 2 μL/min into a triple quadrupole mass spectrometer (Waters Xevo TQ-S). Electrospray ionisation in positive ion mode was used with the following settings: capillary voltage – 3.0 kV; cone voltage – 30 V; ion source temperature – 100°C; collision energy – 20 V. Nitrogen and argon were used as the curtain and the collision gases, respectively.

### 4.15 LC-MS/MS analysis of TPP-IAM or IAM quenched samples

*IAM-quenching*. For MS/MS analysis of samples quenched with IAM, a triple-quadrupole mass spectrometer was used (Waters Xevo TQ-S under positive ion mode: source spray voltage, 3.4 kV; ion source temperature, 150°C). Nitrogen and argon were used as curtain and collision gas, respectively. For LC-MS/MS analyses the mass spectrometer was connected in series to an I-Class ACQUITY UPLC system (Waters). Samples were stored in an autosampler at 8°C and 2 μL samples were injected via a 15 μL flow-through needle and RP-UPLC at 40°C using an I-Class ACQUITY UPLC BEH C18 column (1 × 50 mm, 130 Å, 1.7 μm: Waters) with a Waters UPLC filter (0.2 μm. Waters). MS buffers A (95 % water, 5% ACN, 0.1% FA) and B (90% ACN, 10 % water, 0.1% FA) were infused at 200 μL/min using the following gradient (the proportion of MS solvent B is given in %): 0–0.3 min, 5 %; 0.3–2 min, 5–100 %; 2–2.5 min, 100 %, 2.5–2.8, 100–5 %; 2.8–3.0 min, 5 %. Compounds were detected in multiple reaction monitoring (MRM) in positive ion mode. The peak areas of the molecules were quantified using the MassLynx 4.1 or 4.2 software.

The following MS settings were used for the MRM detection of the individual compounds.

**Table:** 

Molecule	MRM transition	Cone voltage (V)	Collision energy (V)
GS-CAM	365.1596 > 290.0879	32	10
GSS-CAM	397.1596 > 322.0562	26	12
GS-benzoyl	412.1596 > 283.0566	18	6
MitoNAP-SSG (M^2+^)	413.8404 > 183.0482	32	70
MitoNAP-SSG (M^+^)	826.3936 > 262.0814	2	46
MitoNAP-S-CAM	578.3298 > 262.0817	26	38
MitoNAP-SS-CAM	610.2660 > 262.0873	2	30

*TPP-IAM quenching*. For MS/MS analysis, a triple-quadrupole mass spectrometer was used (Waters Xevo TQ-S under positive ion mode: source spray voltage, 2.6 kV; ion source temperature, 150°C). Nitrogen and argon were used as curtain and collision gas, respectively. For LC-MS/MS analyses the mass spectrometer was connected in series to an I-Class ACQUITY UPLC system (Waters). Samples were stored in an autosampler at 8°C and 0.5–5 μL samples were injected via a 15 μL flow-through needle and RP-UPLC at 40°C using an I-Class ACQUITY UPLC BEH C18 column (1 × 50 mm, 130 Å, 1.7 μm; 0.2 μm; Waters Waters) with a Waters UPLC filter (0.2 μm; Waters). MS buffers A (95% water, 5% ACN, 0.1 % FA) and B (90 % ACN, 10 % water, 0.1 % FA) were infused at 200 μL/min using either of the following gradients (the proportion of MS solvent B is given in %): 3 min gradient: 0–0.3 min, 5 %; 0.3–2 min, 5–100 %; 2–2.5 min, 100%, 2.5–2.8, 100–5 %; 2.8–3.0 min, 5 %. 5 min gradient: 0–0.3 min, 5 %; 0.3–3.0 min, 5–100 %; 3.0–4.0 min, 100 %, 4.0–4.1, 100–5 %; 4.1–5.0 min, 5 %. Compounds were detected in multiple reaction monitoring (MRM) in positive ion mode. The peak areas of the molecules were quantified using the MassLynx 4.1 or 4.2 software.

The following MS settings were used for the MRM detection of the individual compounds.

**Table:** 

Molecule	MRM transition	Cone voltage (V)	Collision energy (V)	UPLC
GS-CAM-TPP	695.4149 > 422.2755	38	40	3 or 5 min
GSS-CAM-TPP	727.4149 > 262.1422.	98	40	
GS-benzoyl	412.1596 > 283.0566	18	6	
MitoNAP-SSG (M^2+^)	413.8404 > 183.0482	32	70	
MitoNAP-CAM-TPP	454.9681 > 262.1792	52	32	
MitoNAP-SS-CAM-TPP	470.9681 > 262.0474	50	40	

### 4.16 LC-MS/MS characterization of *in vitro* reaction products

To analyze the reaction in time, the reaction mixture of 100 μM of MitoPerSulf, MitoNAP-SH and different concentration of GSH (0.2 or 1 mM) in 25 mM HEPES buffer (pH 7.8) was incubated at 37°C for 15 min and 50 μL fractions taken after 1, 5, 10 and 15 min and were immediately mixed with 20 mM iodoacetamide (5.5 μL from 200 mM fresh stock solution in water) and incubated at RT in dark for 20 min. Blocked samples were diluted 1:200 with 20% acetonitrile in 0.1 % formic acid and immediately analyzed by LC-MS/MS.

### 4.17 LC-MS/MS characterization of reaction products-in organelle

MitoPerSulf metabolism within mitochondria. RHM (1 mg protein/mL) were incubated with MitoPerSulf in KCl buffer (120 mM KCl, 10 mM HEPES, 1 mM EGTA, 1 mM MgCl_2_ and 5 mM KH_2_PO_4_, pH 7.4) at 37°C, supplemented with succinate (10 mM) and rotenone (4 μg/mL). Aliquots were centrifuged (1 min at 17 000×*g* at RT) at the indicated times and precipitated mitochondrial pellets were rapidly resuspended in 50 μL of 40 mM TPP-IAM (200 mM stock solution in MeOH or DMSO) in 100 mM HEPES buffer (pH 7.8). Samples were vortexed and sonicated in a sonic bath at RT in dark for 20 min. Next, 200 μL of ACN were added and samples were placed at −20°C for 5 min. Samples were centrifuged (10 min at 17 000×*g*) to pellet proteins and 100 μL of supernatant were retrieved and combined with 400 μl of MS-grade H_2_O containing FA (0.1 %). Subsequently, samples were diluted (in 20 % ACN 0.1 % FA) as required and analyzed by LC-MS/MS to assess levels of TPP-IAM quenched reaction products. All experiments were performed in triplicates and data are mean ± s.e.m.

### 4.18 Determining persulfidation of Cofilin-1 by MitoPerSulf via LC-MS

Cysteines (∼160 μM of total thiols) of his-tagged human recombinant cofilin-1 (∼118 μg) were reduced with TCEP (200 μM) for 30 min at 37°C and TCEP was removed by desalting the sample with BioSpin 6 columns (pre-equilibrated with Chelex-100 treated 25 mM HEPES pH 7.4) prior to distribution of the equal amount of desalted protein into individual tubes (calculated: 11 μg/sample). Samples were incubated with 100 μM MitoPerSulf or AP39 (control received ethanol) and 1 mM GSH for 7.5 min in 25 mM HEPES at 37°C in the total volume of 50 μL of 25 mM HEPES pH 7.4 using the 200 μL PCR-grade test tubes to minimize the gas head space volume. Upon incubation, samples were alkylated by addition of 20 mM IAM (from 200 mM stock solution in Chelex-100 treated water) in dark at room temperature for 20 min. After the treatment, samples were precipitated by addition of 50 μl cold MeOH (−25°C) and 12.5 μL of cold CHCl_3_ and centrifugated for 10 min at 4°C. Both MeOH and CHCl_3_ layers were removed from protein precipitates (protein disc in between two liquid phases) and the residual liquid was evaporated on air leaving the precipitated and labelled proteins at the bottom of the tubes. Precipitated proteins were dissolved in 50 μL of 50 mM ammonium bicarbonate buffer pH 7.8 containing 1 mM CaCl_2_ and 12.5 ng/μL trypsin and digested overnight at 37°C.

Peptides were resuspended in 3 % ACN, 0.1 % TFA buffer and portions were fractionated by liquid chromatography on a Biosphere C18 reversed-phase column, 75 μm inner diameter, 100 mm length (Nanoseparations, Nieukoop, Netherlands) in a Proxeon EASY-nLC II system using Buffer A (0.1 % formic acid, 2 % acetonitrile) and Buffer B (98% acetonitrile, 0.1 % formic acid) and a gradient of 2–35 % B over 84 min at a flow rate of 300 nL/min, followed by an increase in acetonitrile concentration to 90 % B over 5 min and re-equilibration with 2 % B within a total time of 102 min. The eluate was transferred in-line to a LTQ Orbitrap XL ETD mass spectrometer (Thermo Scientific, UK).

Peptides were analyzed by positive ion electrospray mass spectrometry in a data-dependent acquisition mode. Up to ten of the most abundant precursor ions with multiple charge states, were selected and fragmented by CID each second. The *m*/*z* values of precursor and up to 10 fragment ions were measured simultaneously in the Orbitrap (400–2000 *m*/*z* scan, resolution of 60 000) and ion-trap analyzers, respectively. A lock mass ion (polysiloxane, *m*/*z* = 445.1200) was used for internal MS calibration. For protein identification the fragment patterns were compared to the UniProt database using the Mascot search engine with Proteome Discoverer (v1.4) software (Thermo Scientific). Relative quantification was performed by comparing the peak area of XICs (extracted ion chromatograms) for the monoisotopic peak using Xcalibur software (Thermo Scientific).

### 4.19 Tag switch assay

Detection of protein persulfidation was performed by using the dimedone-based tag-switch method as reported previously [38] with modifications. In brief, 1 mg of RHM proteins were incubated in 2 mL of KCl buffer with 10 μM MitoPerSulf, MitoNAP or vehicle (EtOH) in the presence of 10 mM succinate and 4 μg/mL rotenone for 5 min at 37 °C. Subsequently, pelleted mitochondria (1 min at 17 000 g) were resuspended in 50 μL of HENS buffer (50 mM HEPES, 1 mM EDTA, 0.1 mM Neocuproine, 1 % NP-40, 2% SDS and protease inhibitor cocktail, pH 7.4) supplemented with 5 mM 4-chloro-7-nitrobenzofurazan (NBF–Cl, from 500 stock solution in DMSO) and incubated at 37°C for 30 min in dark. Proteins were retrieved using methanol/chloroform precipitation (H_2_O/MeOH/CHCl_3_: 4/4/1) and obtained protein pellets were resuspended using ultrasonication in 50 mM HEPES buffer (pH 7.4) containing 1 % SDS. Protein concentration was determined by BCA assay and 1 mg of protein were labelled with 25 μM of Daz-2-Cy5 alkyne click master mix [38] for 30 min at room temperature in the dark. After labelling, protein pellets obtained using methanol/chloroform were resuspended using ultrasonication in 50 mM HEPES buffer (pH 7.4) containing 1 % SDS and equal amount of protein (approximately 50 μg/sample) were resolved using standard Laemmli reducing 10% SDS PAGE. After electrophoresis, gel was fixed in the dark for 30 min, washed and equilibrate with dH_2_O and scanned using Typhoon FLA 9500 fluorescent scanner (Cy3 and Cy5 fluorescence was recorded using 473 and 635 nm filter sets). Obtained raw images were post processed using ImageJ software.

### 4.20 LAD ligation model

We used an open-chest, *in situ* mouse cardiac infarction model as recently described (Prag et al., 2022). Briefly, Wild-type male C57BL/6J mice (8–10 weeks of age; Charles River Laboratories, UK) were anethetized with sodium pentobarbital (70 mg per kg of body weight intraperitoneally (i.p.)), intubated endotracheally and ventilated with 3 cm H_2_O positive-end expiratory pressure. We monitored the adequacy of the anesthesia using corneal and withdrawal reflexes, and additional anesthesia was administered as needed throughout the experiment. We kept the ventilation frequency at 240 breaths per minute with a tidal volume between 125 μL and 150 μL. We performed a small thoracotomy, and the heart was exposed by stripping of the pericardium. All hearts underwent 30 min of regional ischemia by ligation of a main branch of the left coronary artery. We introduce MitoPerSulf or MitoNAP-SH (100 ng per kg body weight each) 10 min before reperfusion as a slow infusion intravenously into a tail vein over 20 min.

We assessed infarct size after 120 min of reperfusion using triphenyltetrazolium chloride (TTC) staining and expressed it as a percentage of the risk zone as described previously (Prag et al., 2022). For various experiments on treated tissues, we removed the left ventricle at various time points after reperfusion, as indicated in the corresponding Fig. legends.

### 4.21 Statistical analyses

Error bars represent the s.e.m. from at least three replicates unless otherwise stated. We quantified P values using Student's *t*-test or one-way ANOVA. Values of P < 0.05 was considered as statistically significant.

## Author contributions

M. P. M., R. C. H., T. K., A. M. J., and J. Lj. M. carried out study conception and design. J. Lj. M. and N. B. designed, performed, and analyzed most experiments. A. L. helped in method development. J. F. M., D. A., T. N., H. A. P., and O. S. carried out *in vivo* and *ex vivo* experiments and tissue sampling. T. K. supervised mouse experiments. T. A. P., T. N. and G. R. B., carried out *in vitro* experiments. N. B. and J. Lj. M. developed, optimized, and performed MRM analysis. J. Lj. M. and J. L. M. generated corresponding fluorescently labelled cell line and performed all microscopy analysis that was supervised by J. P.. J. M. G., S. T. C., A. A. I. N., S. W. and R. C. H. designed and synthesized compounds. The manuscript was written by J. Lj. M and M. P. M. with assistance from all other authors. The study was directed by M. P. M., T. K., and R. C. H.

## Declaration of competing interest

Authors have no conflict of interest to declare.

## Data Availability

Data will be made available on request.

## Abbreviations

IR: ischemia-reperfusion
H_2_S: hydrogen sulfide
RET: reverse electron transport
GYY4137: 4-Methoxyphenyl(morpholino)phosphinodithioate morpholinium salt
AP123: (10-(4-carbamothioylphenoxy)-10- oxodecyl)triphenylphosphonium bromide)
AP39: (10-Oxo-10-(4-(3-thioxo-3H-1,2-dithiol-5- yl)phenoxy)decyl)triphenylphosphonium bromide
HS-NSAIDs: hydrosoluble non-steroidal anti-inflammatory drugs
DATS-MSN: diallyl trisulfide-loaded mesoporous silica nanoparticles
r-SPSH: reversible *S*-thiolation of protein cysteine residues
TPP: triphenylphosphonium cation
GSH: Glutathione
MitoPerSulf: 5-(2′-Acetylamino-3′-benzoyldisulfanyl-3′- methylbutyrylamino)pent-1-yl]- riphenylphosphonium methanesulfonate
MitoNAP-SSH: MitoNAP persulfide
MitoNAP-SS-CAM: carbamidomethylated MitoNAP persulfide
Iodoacetamide: IAM
LC-MS/MS: liquid chromatography tandem mass spectrometry
CAM: carbamidomethylated
GSCOPh: benzoylated glutathione
MitoNAP-SSG: glutathionylated MitoNAP
GSSH: glutathione persulfide
GSS-CAM: carbamidomethylated glutathione persulfide
GS-CAM: carbamidomethylated glutathione
MitoNAP-S-CAM: carbamidomethylated glutathione MitoNAP
Δψ: membrane potential
IAM-TPP: triphenylphosphonium iodoacetamide
MitoNAP-SS-CAM-TPP: TPP-carbamidomethylated MitoNAP persulfide
MitoNAP-S-CAM-TPP: TPP-carbamidomethylated MitoNAP
GSS-CAM-TPP: TPP-carbamidomethylated glutathione persulfide
LAD: left anterior descending
