# Supplementary material for: Rapid and selective generation of H2S within mitochondria protects against cardiac ischemia-reperfusion injury
Source: Redox Biol. 2022 Aug 5;55:102429. doi: 10.1016/j.redox.2022.102429 (PMC9382561; doi:10.1016/j.redox.2022.102429)
Supplement: Multimedia component 1 [file mmc1.docx]

***Supplementary Figures***

**Rapid and Selective Generation of H_2_S within Mitochondria Protects Against Cardiac Ischemia-Reperfusion Injury**

Jan Lj. Miljkovic^a^, Nils Burger^a^, Justyna M. Gawel^b^, John F. Mulvey^c^, Abigail A. I. Norman^b^, Takanori Nishimura^c,d^, Yoshiyuki Tsujihata^d^, Angela Logan^a^, Olga Sauchanka^c^, Stuart T. Caldwell^b^, Jordan L. Morris^a^, Tracy A. Prime^a^, Stefan Warrington^b^, Julien Prudent^a^, Georgina R. Bates^a^, Dunja Aksentijević^e^, Hiran A. Prag^a,c^, Andrew M. James^a^, Thomas Krieg^c^, Richard C. Hartley^b,*^, Michael P. Murphy^a,c,*^

^a^MRC Mitochondrial Biology Unit, University of Cambridge, Cambridge Biomedical Campus, CB2 0XY, UK

^b^School of Chemistry, University of Glasgow, Glasgow, G12 8QQ, UK

^c^Department of Medicine, University of Cambridge, Cambridge, CB2 0QQ, UK

^d^Innovative Biology Laboratories, Neuroscience Drug Discovery Unit, Takeda Pharmaceutical Company Limited, 251-8555, Japan

^e^ Centre for Biochemical Pharmacology, William Harvey Research Institute, Barts and the London School of Medicine and Dentistry, Queen Mary University of London, Charterhouse Square, London, United Kingdom

*Corresponding authors: mpm@mrc-mbu.cam.ac.uk (M.P.M), Richard.Hartley@glasgow.ac.uk (R.C.H.)

*Keywords:* Hydrogen sulfide donors, mitochondria, ischemia-reperfusion injury, mitochondria targeting, reverse electron transport (RET)


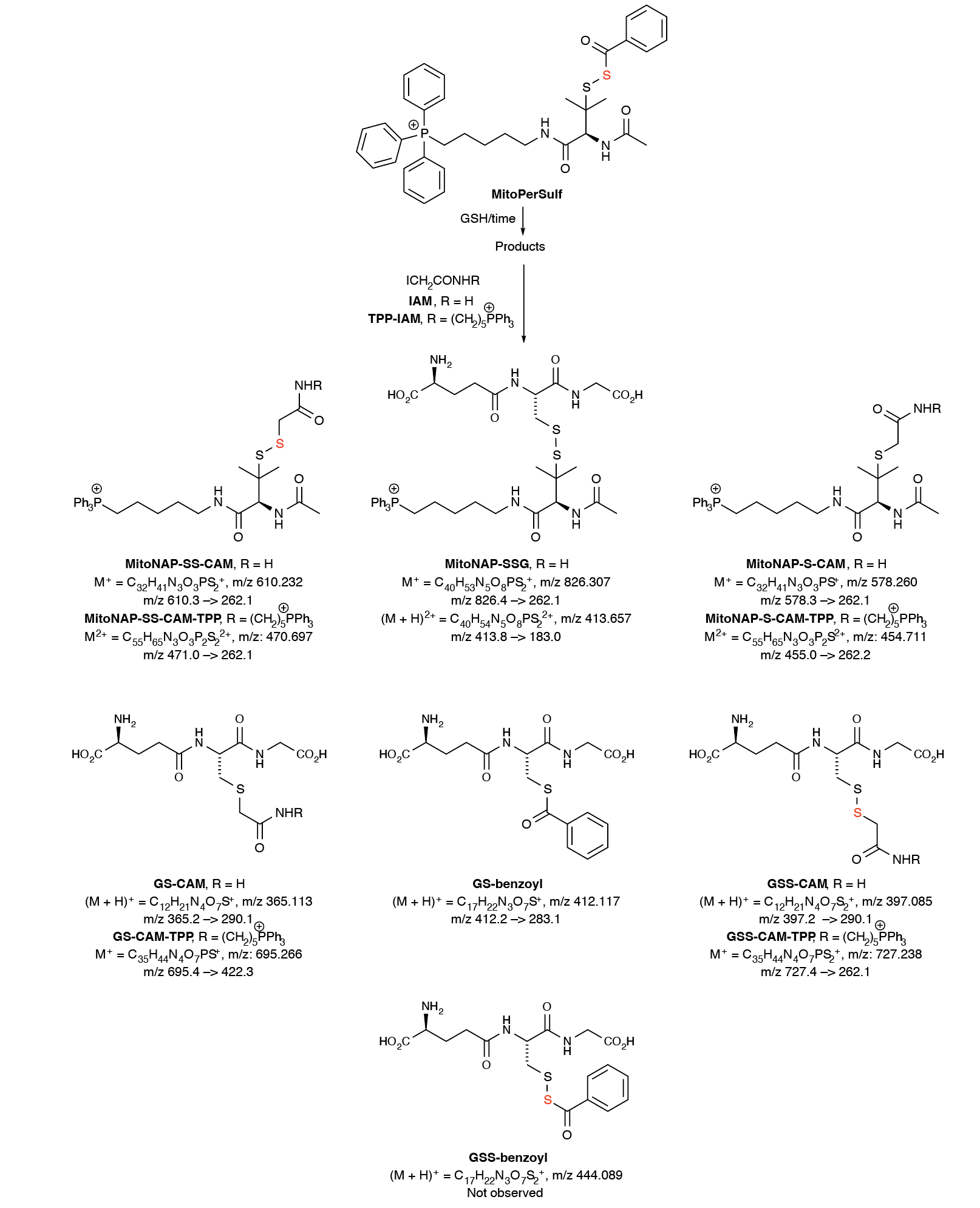


**Fig. S1**. **LC-MS/MS-Based Detection of the Reaction Products of MitoPerSulf and GSH**

Following reaction of MitoPerSulf (100 µM) with GSH (0.2 or 1 mM) for various times samples were blocked with IAM or IAM-TPP and analyzed by LC-MS/MS. The structure and m/z ratio for the carbamidomethylated (CAM) as well as IAM-TPP-labelled (CAM-TPP) adducts of potential reaction intermediates, as well as other products, and the m/z of the fragments used for quantification are shown.

**
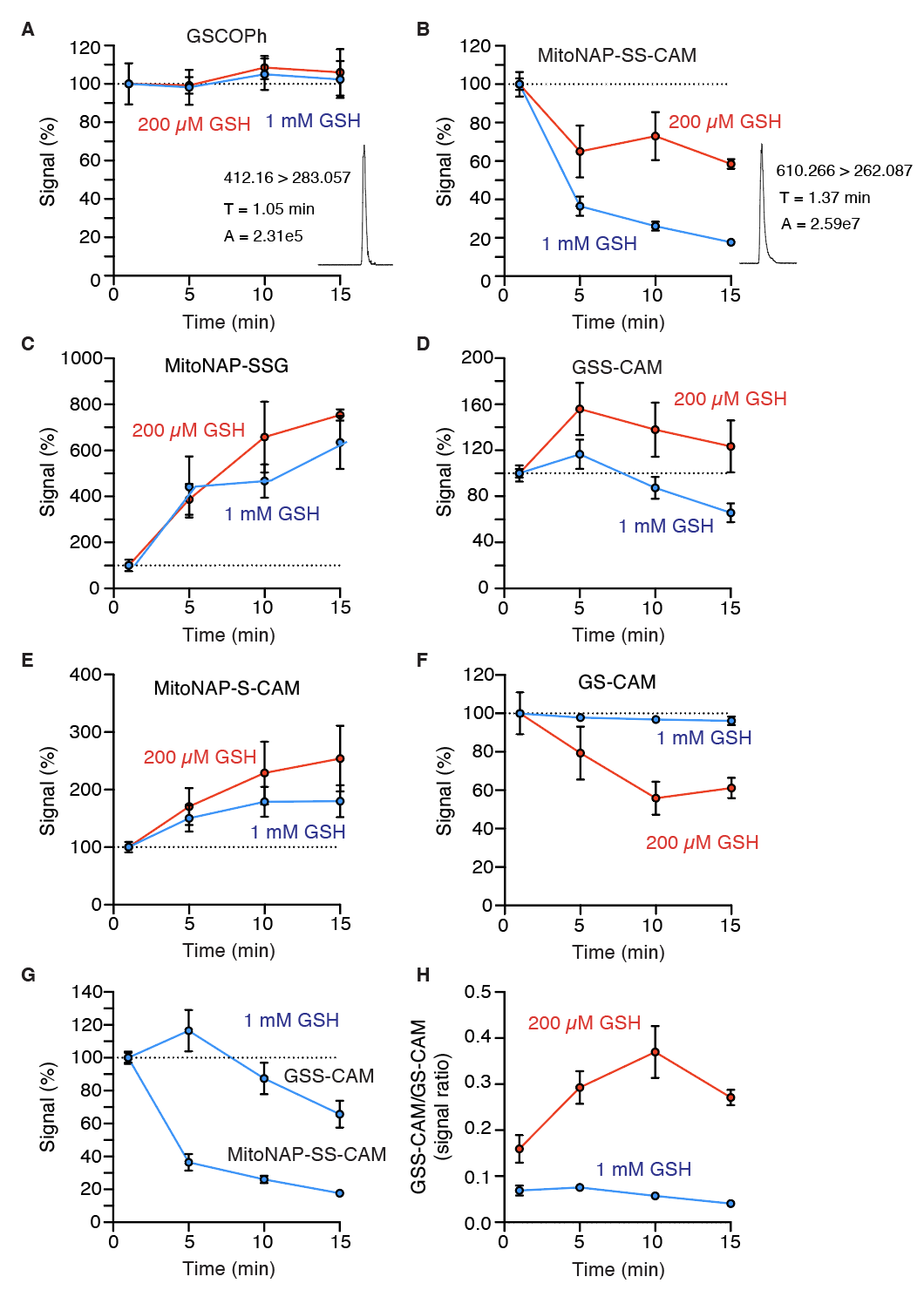
**

**Fig. S2**. **Time Course of Intermediates Formed During Reaction of MitoPerSulf with GSH**

Time-dependent formation of the reaction intermediates described in Figure S1 monitored by LC-MS/MS. Each panel shows the relative peak areas as a percentage of that at the earliest time point for reaction of MitoPerSulf (100 µM) with 1 mM or 0.2 mM GSH followed by quenching with IAM. All experiments were performed in triplicate and data are mean ± s.e.m. n = 6. Representative chromatograms for the 1 min time point are shown for panels A and B, where the transition monitored, elution time (T) and peak area (A) are also indicated.

(A) GSCOPh (m/z = 412.2 > 283.1).

(B) MitoNAP-SS-CAM (m/z = 610.3 > 262.1).

(C) GSS-CAM (m/z = 397.2 > 290.1).

(D) MitoNAP-SSG (m/z = 413.8 > 183.0).

(E) MitoNAP-S-CAM (m/z = 578.3 > 262.1)

(F) GS-CAM (m/z = 365.2 > 290.1).

(G) Amounts of MitoNAP-SS-CAM and GSS-CAM formed upon reaction of MitoPerSulf with 1 mM GSH.

(H) Ratio of the MS signal for glutathione persulfide (GSS-CAM) relative to that for GS-CAM from the reaction of MitoPerSulf (100 µM) with 1 mM or 0.2 mM GSH followed by quenching with IAM. Results are presented as the GSSH/GSH ratio. Results are mean ± s.e.m., n = 5 or 6.

**
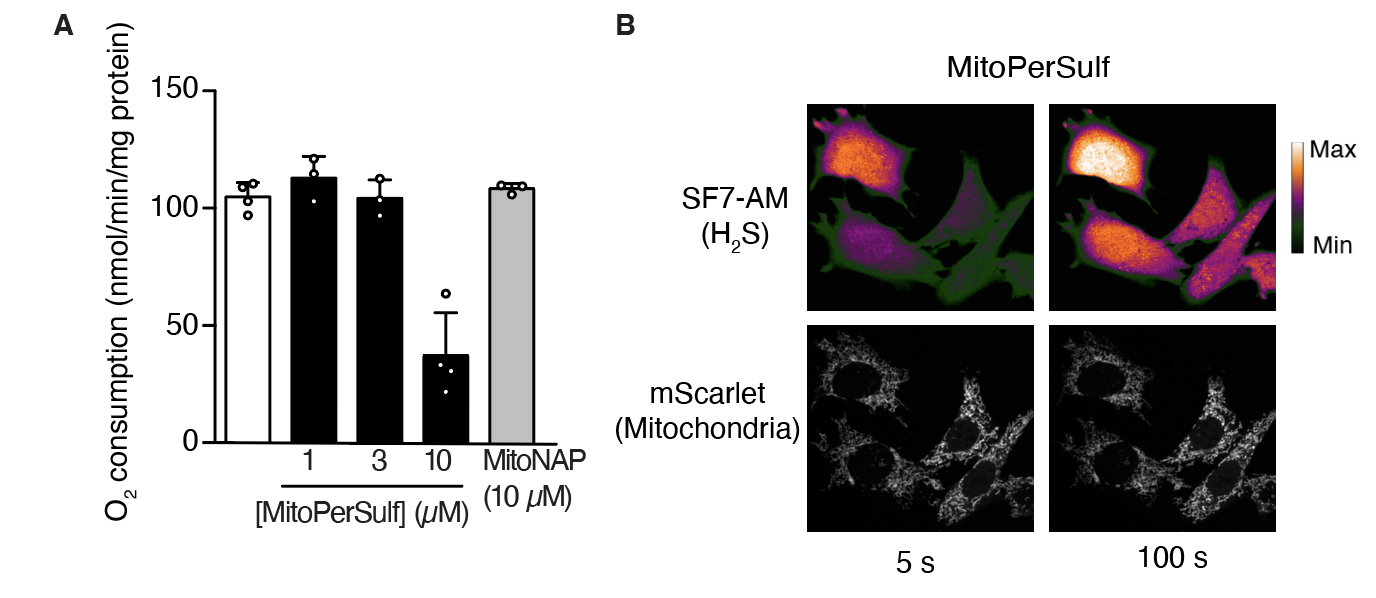
**

**Fig. S3**. **Metabolism of MitoPerSulf within isolated mitochondria**

(A) Effect of MitoPerSulf on respiration of isolated mitochondria. An Oxygraph-2k (O2k, Oroboros instruments, Innsbruck, Austria) was used. Rat heart mitochondria (0.25 mg protein/ml) in KCl buffer (120 mM KCl, 10 mM HEPES, 1 mM EGTA, 5 mM potassium phosphate, 1 mM DTPA and 10 μM neocuproine, pH 7.4, at 37 **°**C supplemented with 0.15% BSA) were incubated for 3 min with 10 mM glutamate/malate and then the indicated amounts of MitoPerSulf or MitoNAP-SH were added and 5 min later ADP (500 µM) was added and respiration rate measured. Data are mean ± SEM of at least three biological replicates.

(B) Formation of H_2_S in mitochondria by MitoPerSulf. Separated channels demonstrating the formation of H_2_S as shown by increasing changes from green to white colour of SF7-AM fluorescence. This is compared with the signal of the stably expressed mitochondria-targeted version of the mScarlet fluorescent protein (monochromatic representation, lower panel).


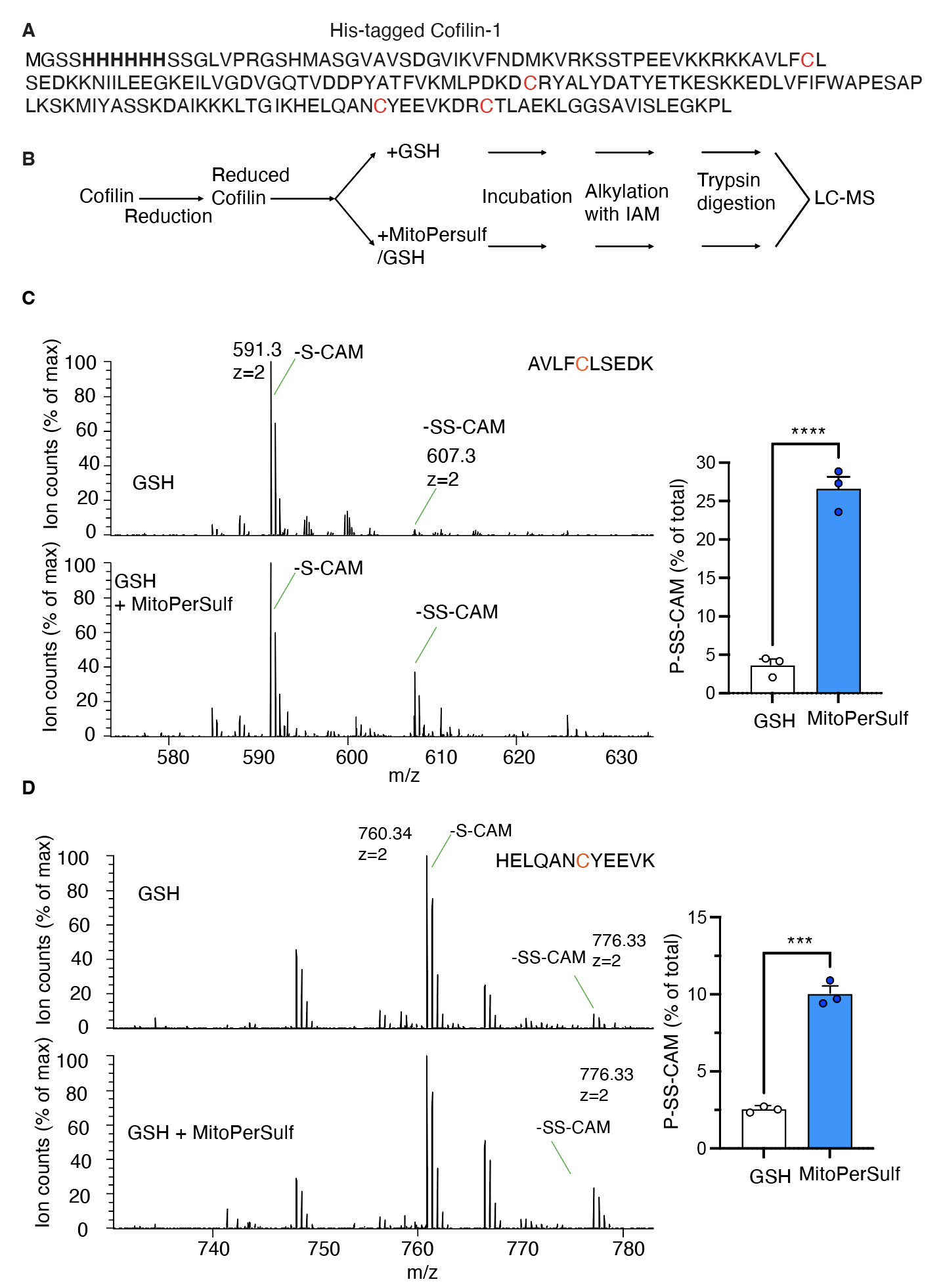


**Fig. S4. Protein persulfidation of Cofilin-1 by MitoSulf *in vitro***

(A) His-tagged Cofilin 1, with the 4 cysteine residues indicated in red.

(B) Schematic for the treatment and analysis of Cofilin-1 with MitoPerSulf. Cofilin-1 (11 µg sample) was reduced with TCEP, treated with 200 μM GSH in the presence or absence of 20 µM MitoPerSulf, then alkylated with IAM. Tryptic peptides were then analyzed by LC-MS for the presence of CAM modified cysteine-containing peptides, or CAM modified persulfidated cysteine-containing peptides. For details see Methods.

(C) Analysis of the Cofilin-1 peptide containing Cys39. Typical MS spectra of the peptide containing the CAM-modified Cys39 or the CAM-modified persulfidated Cys39. The bar graph shows the comparison of the quantification (monoisotopic peak area) of the persulfidated Cys peptide as a % of the total. Data are presented as mean ± s.e.m. (n = 3), **** P < 0.0001, Student’s t-test

(D) Analysis of the Cofilin-1 peptide containing Cys139. Typical MS spectra of the peptide containing the CAM-modified Cys139 or the CAM-modified persulfidated Cys139. The bar graph shows the comparison of the quantification (monoisotopic peak area) of the persulfidated Cys peptide as a % of the total. Data are presented as mean ± s.e.m. (n = 3), *** P < 0.001, Student’s t-test

**Fig. S5. Persulfidation of mitochondrial proteins.**

Rat heart mitochondria (1mg protein) were resuspended in 2 mL KCl buffer supplemented with rotenone (4 μg/mL) and succinate (10 mM), and treated for 10 min with various concentrations of MitoPerSulf or MitoNAP. After treatment, samples were centrifuged and mitochondrial pellets were processed using the dimedone-based tag switch assay. Representative images demonstrated fluorescence in gel-based detection of persulfidated mitochondrial proteins (left image) and total mitochondrial protein load (right image) of same samples. Original raw grayscale images were presented using artificial colour masks.


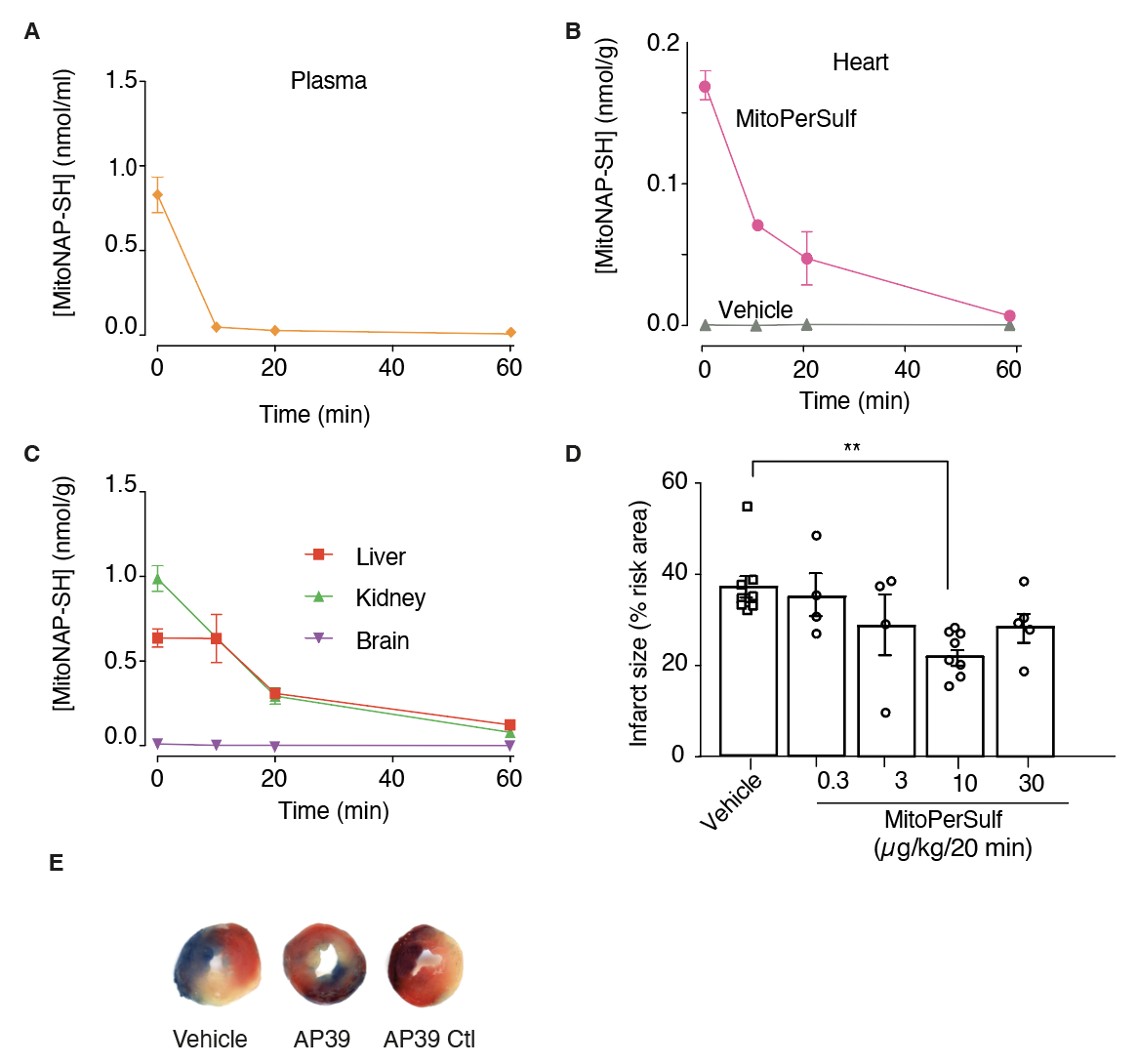


**Fig. S6. The distribution of MitoPerSulf and its derivatives *in vivo***.

The distribution of MitoPerSulf was assessed within mouse tissues after a single i.v. tail vein injection of 0.2 mg/kg of MitoPerSulf in 100 μL saline in wild-type male C57BL/6J mice and accumulation of MitoNAP-SH in plasma and in each tissue was analyzed by LC-MS/MS spectrometry. Two mice were injected for each time point and data are means ± range.

MitoPerSulf and its derivatives inside the tissues were reduced by DTT to MitoNAP-SH extracted and analyzed.

(A) Pharmacokinetics of MitoPerSulf and its derivatives in plasma.

(B) Pharmacokinetics of MitoPerSulf and its derivatives in the heart compared with vehicle.

(C) Pharmacokinetics of MitoPerSulf and its derivatives in liver, kidney and brain.

(D) Dose-response for protection against cardiac IR injury by MitoPerSulf. Mice were subjected to 30 minutes ischemia by ligation of the left anterior descending coronary artery (LAD) ligation followed by 120 minutes of reperfusion. MitoPerSulf or vehicle (0.5% DMSO) were infused into the tail vein at 5 μL/min for 20 min starting 10 min before reperfusion. Myocardial infarct size was then determined as a percentage of the area at risk quantified from a single mouse by triphenyl tetrazolium chloride (TTC) stain. **p<0.01, one-way ANOVA, n = 4-8 ± s.e.m.

(E) Representative TTC stains of mouse hearts from the experiment reported in Fig. 5C.

**
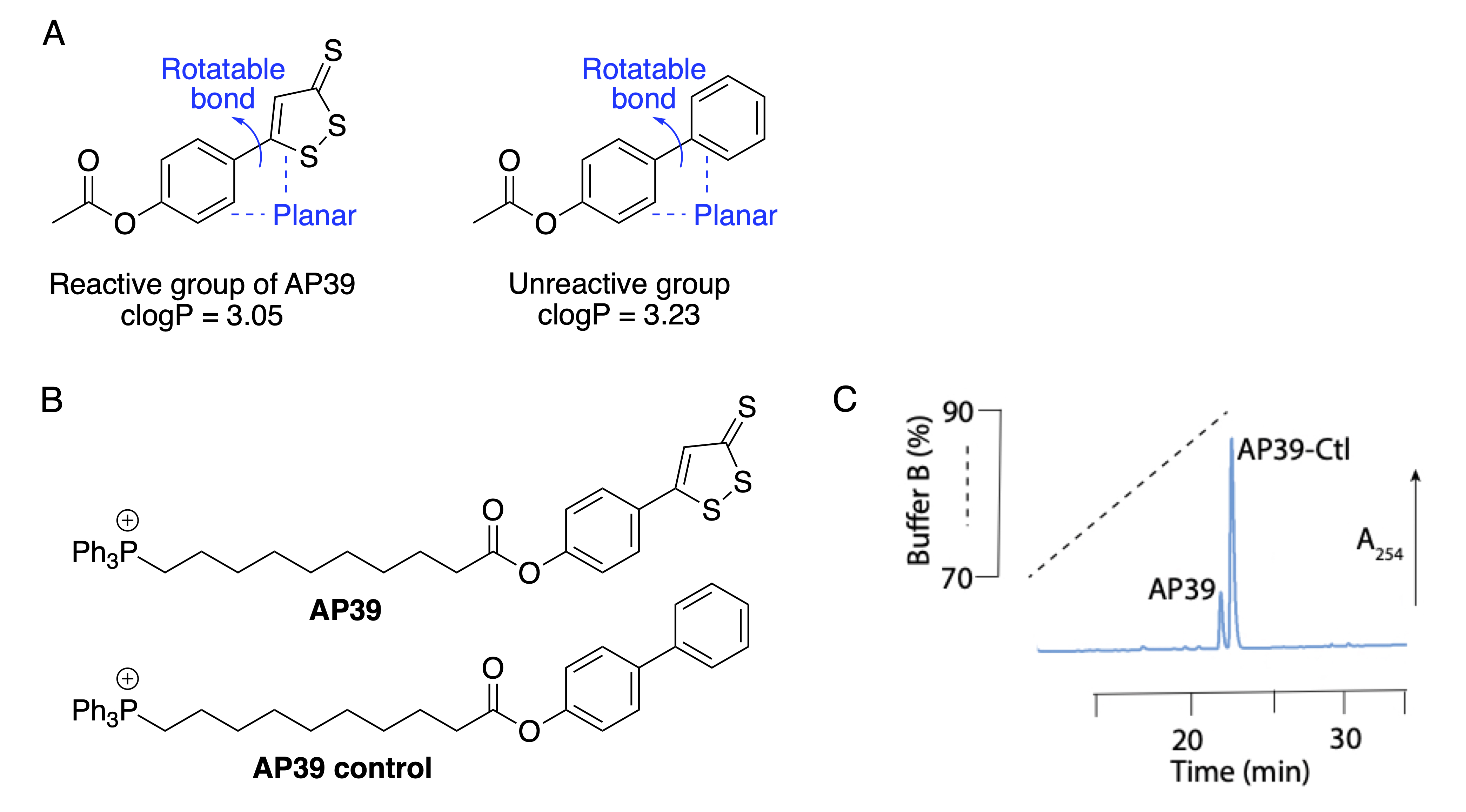
**

**Fig. S7. The hydrophobicity of AP39 and AP39 control**

(A) The reactive 1,2-dithio-3-thione head group of AP39 and that of the corresponding phenyl analogue are shown, along with their calculated clogPs.

(B) The structures of AP39 and the AP39 control.

(C) RP-HPLC of AP39 and AP39 control. The mobile phase was water + 0.1% (v/v) trifluoroacetic acid (TFA) (Buffer A) and acetonitrile + 0.1% (v/v) TFA (Buffer B). Sample (900 µl, containing 10 nmol AP39 and 10 nmol AP 39 Ctrl in 40% acetonitrile + 0.1% (v/v) TFA) were separated using the following gradient: 0-2 min: 5% buffer B, 2 - 4 min: 49% buffer B, 4 - 26 min: 100% buffer B, 26-31 min:100% buffer B, 31- 36 min: 100% - 5% buffer B, 36 - 40 min: 5% buffer B. Samples were detected at 254 nm and peaks identified by comparing the retention times of the known compounds.

**Table S1. Reagents and resources table**

| **REAGENTS or RESOURCE** | **SOURCE** | **IDENTIFIER** |
| --- | --- | --- |
| **Chemicals, solvents and recombinant proteins** |  |  |
| Chelex-100 sodium form | Sigma Aldrich | #C7901; 11139-85-8 |
| SF7-AM | Cayman Chemicals | #14623; 1416872-50-8 |
| WSP-5 | Cayman Chemicals | #16929; 1593024-78-2 |
| Hexadecyltrimethylammonium bromide | Sigma Aldrich | #H6269; 57-09-0 |
| DMSO | Sigma Aldrich | #276855; 67-68-5 |
| AP39  (note that residual CH_2_Cl_2_ was removed under vacuum before use) | Cayman Chemicals | #17100; 1429173-57-8 |
| 2,2’-Dithiobis(benzothiazole) | Sigma Aldrich | #D218154; 120-78-5 |
| DMEM cell culture medium with high glucose, GLUTAMAX and pyruvate | ThermoFisher Scientific | #31966-021 |
| 4-(2-Hydroxyethyl)piperazine-1-ethanesulfonic acid (HEPES) | Sigma Aldrich | #H3375; 7365-45-9 |
| Dulbecco’s Phosphate Buffer Saline | ThermoFisher Scientific | #14190-094 |
| L-Glutathione (Reduced) | Sigma Aldrich | #G4251; 70-18-8 |
| Lead (II) acetate trihydrate | Sigma Aldrich | #L-3396; 6080-56-4 |
| Foetal Bovine Serum | ThermoFisher scientific | #10270-106; 9014-81-7 |
| Streptomycin-Penicillin | Sigma Aldrich | #P4458 |
| Hygromycin B | Roche | #10843555001; 31282-04-9 |
| 0.25 % Trypsin EDTA (1x) Solution | ThermoFisher Scientific | #25200072 |
| 2-Amino-2-(hydroxymethyl)-1,3-propanediol | Sigma Aldrich | #93350; 77-86-1 |
| (Ethylenedinitrilo)tetraacetic acid | Sigma Aldrich | #03609; 60-00-4 |
| Sucrose | Sigma Aldrich | #S0389; 57-50-1 |
| Bovine Serum Albumin | Sigma Aldrich | #A3803; 9048-46-8 |
| Potassium chloride | Fisher Scientific | #P/4280/53; 7447-40-7 |
| Magnesium (II) chloride hexahydrate | Sigma Aldrich | #63064; 7791-18-6 |
| Potassium dihydrogen orthophosphate | Fisher Scientific | #P/4800/53; 7778-77-0 |
| Amplex UltraRed | ThermoFisher | #A36006; 119171-73-2 |
| Carbonyl cyanide 4-(trifluoromethoxy)phenylhydrazone | Sigma Aldrich | #C2920; 370-86-5 |
| Horse radish peroxidase | Sigma Aldrich | #P8250; 9003-99-0 |
| Superoxide dismutase | Sigma Aldrich | #S8160; 9054-89-1 |
| Acetonitrile | ROMIL Pure Chemistry | #H049L; 75-05-8 |
| Trifluoracetic acid | ThermoFisher Scientific | #85183; 76-05-1 |
| Succinic acid | Sigma Aldrich | #398055; 110-15-6 |
| Polybrene | Merck Millipore | #TR-1003; 28728-55-4 |
| Ethanol absolute | VWR | #20821.330; 64-17-5 |
| Methanol | Fisher Scientific | #M/4062/17; 67-56-1 |
| Dichloromethane | Sigma Aldrich | #676853; 75-09-2 |
| Chloroform | Sigma Aldrich | #650471; 67-66-3 |
| 1.4-Dithiothreitol | Roche | #35085929; 3483-12-3 |
| Tris(2-carboxyethyl)phosphine hydrochloride | Sigma Aldrich | #646547; 51805-45-9 |
| Iodoacetamide | Sigma Aldrich | #I1149; 144-48-9 |
| Formic acid |  | #00940; 64-18-6 |
| 2,3,5-Triphenyltetrazolium chloride | Sigma Aldrich | #T8877; 298-96-4 |
| Rotenone | Santa Cruz Biotechnology | #203242; 83-79-4 |
| Na_2_S anhydrous | Sigma Aldrich | #407410; 1313-82-2 |
| Trypsin proteomic grade | Roche | #11418475001 |
| Ammonium bicarbonate | Sigma Aldrich | #40867; 1066-33-7 |
| Recombinant Human Cofilin-1 | Abcam | #62958 |
| **PmeI** | New England BioLabs | #R0560L |
| **BamHI** | New England BioLabs | #R3136L; 81295-09-2 |
| **T4 DNA Ligase** | New England BioLabs | #M0202M; 232-770-0 |
| TPP-IAM | University of Glasgow, UK | Prepared by the literature method [63] by A. Norman under direction of R. C. Hartley. |
| AP39 Control | This paper |  |
| (9-carboxynonyl)triphenylphosphonium bromide | University of Glasgow, UK | Prepared for this study by S. T. Caldwell following the literature method [74] under the direction of R. C. Hartley |
| MitoPerSulf Mesylate | This paper |  |
| MitoNAP-SH Mesylate  [originally termed MitoNAP (Prime et al., 2009)] | University of Otago, NZ,  University of Glasgow, UK | The first batch was prepared at the University of Otago under the direction of R. A. J. Smith; the main batch for this study was prepared by A. Norman under the direction of R. C. Hartley. Both used the literature method [56]. |
| 4-Phenylphenol | Fluorochem | #094138; 92-69-3 |
| 4-Dimethylaminopyridine (DMAP) | Alfa Aesar | #A13016; 1122-58-3 |
| *N*-(3-Dimethylaminopropyl)-*N*’-ethylcarbodiimide hydrochloride (EDCI) | Fluorochem | #024810; 25952-53-8 |
| 4-chloro-7-nitrobenzofurazan | Sigma Aldrich | #163260; 10199-89-0 |
| DAz-2 | Cayman Chemical | # 13382; 1176905-54-6 |
| Cyanin5 alkyne | Luminprobe | #A30B0; 1223357-57-0 |
| Copper(II)-TBTA complex | Luminprobe | #21050; |
| Ascorbic acid | Sigma Aldrich | #A5960; 50-81-7 |
| Protease inhibitor cocktail | Sigma Aldrich | # 11697498001 |
| Neocuproine | Sigma Aldrich | #N1626; 332360-00-6 |
| NP-40 | Sigma Aldrich | #492016; 9016-45-9 |
| **Experimental models: cell lines and mice strains** |  |  |
| **Mouse embryonic fibroblast (MEF)** | ATCC | #SCRC-1040 |
| **Human embryonic kidney cells (HEK293T)** | ATCC | #CRL-3216 |
| C57BL/6J | Charles Rivers Laboratories |  |
| Wistar Female Rat | Charles Rivers Laboratories |  |
| **Plasmids, primers and oligonucleotides** |  |  |
| **psPAX2** | Addgene | #12260; http://n2t.net/addgene:12260; RRID: Addgene_12260 |
| **pMD2.G** | Addgene | #12259; http://n2t.net/addgene:12259; RRID: Addgene_12259 |
| **pMTS_mScarlet_N1** | Addgene | #85057; http://n2t.net/addgene:85057; RRID: Addgene_85057 |
| BamHI-MTS SCARLET Antisense  5’-ATATGGATCCTCACTTGTACAGCTCGTCC-3’ | Sigma Aldrich |  |
| PmeI-MTS SCARLET Sense  5’-ATATGTTTAAACATGTCCGTCCTGACGCCG-3’ | Sigma Aldrich |  |
| **Critical commercial assays, reagents and consumables** |  |  |
| Micro Bio-Spin P-6 columns | Bio Rad | #7326221 |
| Soft Tissue Homogenising Mix tubes | OMNI International | #19-627 |
| 0.2 mm glass bottom 96-well plate | CORNING | #4580 |
| 0.22 μm PVDF filter | Merc Millipore | #F2500-13 |
| Whiteman filter paper | Millipore | #3030-917 |
| Pierce BCA Protein Assay Kit | ThermoFisher Scientific | #23225 |
| FuGENE HD Transfection Reagent | Promega | #E2311 |
| 35 mm high µ-Dish | Promega | #81158 |
| Filter paper | Whatman | 3030-917 |
| **Equipment and software** |  |  |
| Precellys 24 Tissue Homogenizer | Bertin Technologies | P000669-PR240-A |
| Oxygraph-2k | Oroboros instruments |  |
| C18 column | Phenomenex | Jupiter 300 Å |
| Widepore C18 guard column | Phenomenex |  |
| 321 Pumps | Gilson |  |
| UV/VIS 151 spectrophotometer | Gilson |  |
| Spectra MAX Gemini XS platereader | Molecular Devices |  |
| Spectrofluorometer RF-5301 PC | SHIMADZU |  |
| Spectra max PLUS 384 | Molecular Devices |  |
| Apollo 4000 free radical analyser | WPI |  |
| 5 mm H_2_S sensor | WPI | #ISO-H2S-100-CXX (5MM) |
| Multi-Port Measurement Chamber | WPI | #NOCHM-4 |
| Xevo TQ-S (triple-quadrupole mass spectrometer) | Waters |  |
| ACQUITY UPLC system | Waters |  |
| ACQUITY UPLC BEH C18 column | Waters |  |
| Bruker AVIII 400 and AVIII 500 NMR spectrometers | Bruker |  |
| MicroTOFq for low and high resolution ESI^+^ | Bruker |  |
| Isolera One Flash Chromatography system using Biotage SNAP Ultra, Biotage KIP or Agela silica gel cartridges | Biotage |  |
| FTIR-8400S spectrometer | Shimadzu |  |
| Bio Scanner V850 | Epson |  |
| Typhoon FLA 9500 | GE Healthcare |  |
| DragonFly Spinning Disc Confocal Microscope | Andor | DFLY-0130 |
| ImageJ | NIH | https://imagej.nih.gov/ij/ |
| MassLynx 4.1 or 4.2 software | Waters | https://www.waters.com/waters/en_US/MassLynx-MS-Software/nav.htm?cid=513662&locale=en_US |
| GraphPad Prism | GraphPad software Inc | https://www.graphpad.com/scientific-software/prism/ |
